# Development of a novel multi‑epitope vaccine against the pathogenic human polyomavirus V6/7 using reverse vaccinology

**DOI:** 10.1186/s12879-024-09046-0

**Published:** 2024-02-09

**Authors:** Reza Salahlou, Safar Farajnia, Nasrin Bargahi, Nasim Bakhtiyari, Faranak Elmi, Mehdi Shahgolzari, Steven Fiering, Srividhya Venkataraman

**Affiliations:** 1 Biotechnology Research Center, Tabriz University of Medical Sciences, Tabriz, Iran; 2 Student Research Committee, Tabriz University of Medical Sciences, Tabriz, Iran; 3 Drug Applied Research Center, Tabriz University of Medical Sciences, Tabriz, Iran; 4 Department of Medical Biotechnology, Faculty of Advanced Medical Sciences, Tabriz University of Medical Sciences, Tabriz, Iran; 5 Department of Microbiology and Immunology, Geisel School of Medicine, and Dartmouth Cancer Center, Lebanon, NH USA; 6 Department of Cell and Systems Biology, University of Toronto, Toronto, ON Canada

**Keywords:** Human polyomavirus, Vaccine, Immunoinformatics, Molecular docking, Immunotherapy

## Abstract

**Background:**

Human polyomaviruses contribute to human oncogenesis through persistent infections, but currently there is no effective preventive measure against the malignancies caused by this virus. Therefore, the development of a safe and effective vaccine against HPyV is of high priority.

**Methods:**

First, the proteomes of 2 polyomavirus species (HPyV6 and HPyV7) were downloaded from the NCBI database for the selection of the target proteins. The epitope identification process focused on selecting proteins that were crucial, associated with virulence, present on the surface, antigenic, non-toxic, and non-homologous with the human proteome. Then, the immunoinformatic methods were used to identify cytotoxic T-lymphocyte (CTL), helper T-lymphocyte (HTL), and B-cell epitopes from the target antigens, which could be used to create epitope-based vaccine. The physicochemical features of the designed vaccine were predicted through various online servers. The binding pattern and stability between the vaccine candidate and Toll-like receptors were analyzed through molecular docking and molecular dynamics (MD) simulation, while the immunogenicity of the designed vaccines was assessed using immune simulation.

**Results:**

Online tools were utilized to forecast the most optimal epitope from the immunogenic targets, including LTAg, VP1, and VP1 antigens of HPyV6 and HPyV7. A multi-epitope vaccine was developed by combining 10 CTL, 7 HTL, and 6 LBL epitopes with suitable linkers and adjuvant. The vaccine displayed 98.35% of the world's population coverage. The 3D model of the vaccine structure revealed that the majority of residues (87.7%) were located in favored regions of the Ramachandran plot. The evaluation of molecular docking and MD simulation revealed that the constructed vaccine exhibits a strong binding (-1414.0 kcal/mol) towards the host's TLR4. Moreover, the vaccine-TLR complexes remained stable throughout the dynamic conditions present in the natural environment. The immune simulation results demonstrated that the vaccine design had the capacity to elicit robust immune responses in the host.

**Conclusion:**

The multi-parametric analysis revealed that the designed vaccine is capable of inducing sustained immunity against the selected polyomaviruses, although further in-vivo investigations are needed to verify its effectiveness.

**Supplementary Information:**

The online version contains supplementary material available at 10.1186/s12879-024-09046-0.

## Background

Polyomaviruses are small, nonenveloped DNA viruses, which are widespread in nature. Non-enveloped polyomaviruses are capable of infecting mammals and birds with their small circular double-stranded DNA genomes of approximately 5.0 kbp [1, 2]. Two major regulatory proteins are encoded by PyVs, the large tumor antigen (LT-ag) and the small tumor antigen (sT-ag), as well as several structural proteins (VP1 and VP2) [3]. Heterologous animal models indicate that PyVs may carry strong oncogenes, that contribute to cancer in humans. Regulatory proteins are important in viral replication and transcription early in the infection cycle, while structural proteins participate in capsid formation later [4].

Among 12 identified human polyomaviruses (HPyVs), six strains are involved in human diseases, especially in different human cancers. HPyV6, HPyV7, Merkel cell polyomavirus (MCPyV), trichodysplasiaspinulosa virus (TSPyV), HPyV9, MWPyV and BK virus (BKV) and JC virus (JCV), as well as newly identified viruses such as KI (KIPyV), WU (WUPyV), are the most commonly identified strains of human polyomavirus (HPyV) [5–7].

The World Health Organization reported increased incidence of skin cancer in the past few decades, with about 8,500 new cases of skin cancer reported daily in the United States. The relationship between HPyV6 and HPyV7 in human skin cancer has recently been elucidated, in recent decade [7, 8]. Several studies have been conducted to confirm the presence of HPyV6- and HPyV7-DNA in cutaneous (Table 1) and non-cutaneous (Table 2) malignancies, including malignant melanoma (MM) [9], non-melanoma skin cancer tissues, basal cell carcinoma (BCC) and squamous cell carcinoma (SCC). Studies on keratoacanthoma and trichoblastoma revealed the presence of HPyV6 in tumors [10]. HPyV7-DNA was found to be detected more frequently in non-cutaneous cancers compared to HPyV6-DNA, whereas HPyV6-DNA was more commonly observed in skin malignancies. A study concluded that all age groups and genders are infected with HPyV6 and 7, and 52–93% of humans are seropositive for HPyV6 while 33–84% are seropositive for HPyV7 [7]. PCR investigations demonstrated HPyV6 and HPyV7 DNA from the skin of both healthy individuals and those experiencing different types of skin tumors. Small and large T antigens are encoded by polyomaviruses, making them potentially oncogenic [11]. Severe cases of HPyV6 and 7 infections are associated with skin disorders, characterized by a significant increase in viral load, expression of dyskeratotic keratinocytes, and the presence of encapsidated virions observed through electron microscopy and sequencing [12]. Moreover, HPyV6 was identified in various forms of epithelial neoplasms, while HPyV7 was observed in thymic epithelial tumors. These discoveries imply that HPyV6 and HPyV7 could be crucial contributors to the development of inflammatory skin conditions and may also possess oncogenic properties [13]. These findings indicate that HPyV6 and HPyV7 may have a crucial involvement in the development of inflammatory skin conditions and potentially possess oncogenic properties [13].

**Table 1:** HPyV-6 and HPyV-7 seroprevalence in primary cutaneous malignancies

Cancer type	HPyV6 Positive	HPyV7 Positive	Reference
Verrucous keratosis	75%	75%	[14]
Squamous cell carcinoma in chronic lymphocytic leukemia cases who had bone marrow transplantation	33.3%	Not determined	[15]
Squamous cell carcinoma	36.4%	54.5%	[14]
Squamous cell carcinoma	100%	100%	[9]
Keratoacanthoma	42.3%	Not applicable	[10]
Trichoblastoma	22.2%	Not applicable	[10]
Cutaneous T-cell lymphomas	20%	8.6%	[16]

(Adapted from [7])

**Table 2:** HPyV6 and HPyV7 prevalence in non-cutaneous human malignancies

Tumor type	HPyV6 Positive	HPyV7 Positive	Reference
Laryngeal squamous cell carcinoma	14.3%	Not detected	[17]
Thymic epithelial tumors	Not detected	62.2%	[18]
Cholangiocarcinoma	9.5%	45.2%	[19]

(Adapted from [7])

It has shown that HPyV6 and 7 bind, and inactivate p53 resulting in tumor progression. P53 suppresses tumor growth by regulating gene expression in response to stressors, such as DNA damage, leading to apoptosis and cell cycle arrest [20]. The transactivation domain of P53 is repressed by interaction with LT-antigen preventing its binding to DNA, eventually leading to cancer in humans [21, 22].

The lack of vaccine against HPyVs might be related to the complexity of the virus and the ability of HPyVs to evade the host immune system by various strategies, such as downregulating the expression of major histocompatibility complex (MHC) molecules, interfering with interferon signaling, and modulating the activity of immune cells [23]. Multi-epitope vaccines contain multiple antigenic fragments (epitopes) from different proteins of the target pathogen. This type of vaccine has several advantages over traditional vaccines, such as inducing a broader and more robust immune response, reducing the risk of antigenic escape and cross-reactivity, and facilitating the production and delivery of the vaccine. Hence, a multi-epitope vaccine against HPyVs could be beneficial for preventing or treating HPyV-associated diseases, especially in immunocompromised patients who are more susceptible to viral reactivation and complications [24].

In recent years, cancer vaccines have shown promising results against different cancers but vaccine development using traditional approaches is complex and requires a significant amount of effort [25]. Compared to traditional laboratory approaches, immunoinformatics enables rapid development of a multi-epitope vaccine, increasing efficiency and reducing costs [26, 27]. Epitope-based peptide vaccines demonstrated effectiveness in providing protective immunity against various viruses including Zika, dengue, SARS-CoV-2, and Coxsackie B viruses [28]. Hence, it is supposed that a peptide-based vaccine against oncogenic polyomavirus could provide an efficient protective vaccine against HPyV6/V7 oncogenic virus strains.

To develop a multi-epitope vaccine, CTL, HTL, and LBL epitopes of the HPyV6 and HPyV7 oncoproteins including large T antigen (LTAg), VP1, and VP2 were identified and the vaccine's stability and effectiveness were analyzed by immunoinformatics methods. The study yielded compelling evidence supporting the likelihood that the multi-epitope vaccine can effectively initiate a strong anti-HyPV immune response.

## Methods

### Retrieval and analysis of protein sequences

Human polyomaviruses 6 and 7 were obtained from the National Center for Biotechnology Information (NCBI) database (https://www.ncbi.nlm.nih.gov/) [29]. Complete amino acid sequence retrieval was performed from the UniProt database (https://www.uniprot.org/) [30] in FASTA format. In addition, the VaxiJen v2.0 server (http://www.ddg-pharmfac.net/vaxijen/VaxiJen/VaxiJen.html) was used to evaluate the antigenicity using the default threshold for viruses. VaxiJen 2.0 server is based on auto and cross-covariance (ACC) transformation methods with 70 − 89% accurate prediction [31, 32]. Then, the AllergenFP v1.0 server (https://ddg-pharmfac.net/AllergenFP/) was employed to find the allergenicity of the proteins. This server uses a novel alignment-free descriptor-based fingerprint approach that produces 88.9% accuracy in the prediction result [33]. TMHMM v2.0 server (https://services.healthtech.dtu.dk/services/TMHMM-2.0/), based on the hidden Markov model (HMM), was utilized for transmembrane (TM) helix prediction [34]. The next step of research included structural proteins that are non-allergenic, antigenic, and display less TM helices.

### Identification and evaluation of Cytotoxic T-Lymphocyte (CTL) epitopes

The important role of cytotoxic T lymphocytes (CTLs) in the host defense mechanism is known [35]. CTLs have a receptor called CD8, which attaches to a molecule called MHC class I on the surface of infected cells. This enables them to deliver molecules that destroy the infection [36]. The NetCTL v1.2 server(https://services.healthtech.dtu.dk/services/NetCTL-1.2/) using weight matrix and artificial neural networks is highly efficient for epitopes prediction of 9-mer CTLs against 12 supertypes including A1, A2, A3, A24, A26, B7, B8, B27, B39, B44, B58, and B62. Using a threshold value of 0.90 to maintain a specificity and sensitivity of 0.98 and 0.74 respectively, this server was used to predict CT epitopes with high combination scores among the obtained protein sequences [37]. The MHC-I binding tool of the IEDB resource was utilized to determine MHC-I binding alleles for each CTL epitope dependent on the CONSENSUS method (http://tools.iedb.org/mhci/) [38]. To characterize antigenicity for individual CTL epitopes of the VaxiJen v2.0 server (http://www.ddg-pharmfac.net/vaxijen/VaxiJen/VaxiJen.html) [31], the allergenic profile of the AllerTOP v2.0 server (https://www.ddg-pharmfac.net/AllerTOP/) [39], toxicity prediction of ToxinPred server (http://crdd.osdd.net/raghava/toxinpred/) [40], and immunogenicity of IEDB Class I Immunogenicity tool (http://tools.iedb.org/immunogenicity/) [41] were used respectively. To separate allergens from non-allergens with a prediction accuracy of 85.3% in five-fold cross-validation, the AllerTop v2.0 server was used, which applies the amino acid descriptors, ACC transformation methods, and k-nearest neighbor (kNN) methods [39]. The ToxinPred server is used for evaluation properties of different peptides using support-vector machines (SVM), that is a machine learning approach combined with a quantitative matrix for toxicity prediction [40]. To confirm whether a specific epitope elicits an immune response or not, immunogenicity prediction was performed. CTL epitopes were utilized for the vaccine construction that were antigenic, non-allergenic, non-toxic, and immunogenic and showed high C-scores.

### Identification and analysis of HTL (Helper T-Lymphocyte) epitopes

Antigen recognition by Helper T cells activates CTLs and B cells to eliminate infectious pathogens [35]. We used the MHC class II binding website of the IEDB resource (http://tools.iedb.org/mhcii/) to predict 15-mer HTL epitopes from the target protein sequences [42]. We also used the CONSENSUS method for predicting protein binding alleles with percentile rank threshold ≤ 2 to maintain consistency [43]. To predict characteristics of individual HTL epitopes we used the VaxiJen v2.0 server (http://www.ddg-pharmfac.net/vaxijen/VaxiJen/VaxiJen.html) [31], ToxinPred server (http://crdd.osdd.net/raghava/toxinpred/) [40], and AllergenFP v1.0 server (https://ddg-pharmfac.net/AllergenFP/), respectively [33]. HTL epitopes were assessed based on their non-toxicity, antigenicity, and non-allergenicity, and considering their cytokine induction ability. IFN-γ plays a major role in inhibiting virus replication by stimulating immune responses of natural killer cells and macrophages, as well as by increasing T cell responses [44]. Interferon-gamma (IFN-γ) prediction was performed by the IFNepitope tool (http://crdd.osdd.net/raghava/ifnepitope/predict.php) with 81.39% precision. We applied the IL4pred tool (https://webs.iiitd.edu.in/raghava/il4pred/) with a threshold score of 0.2 to evaluate the induction of interleukin 4 (IL-4). This operation was executed using SVM-based methods with 75.76% accuracy [45]. Then, the ability to induce both cytokines was prioritized in the selection of HTL epitopes for vaccine construction. For the proteins that were without cytokine-inducing functions, we prioritized the IFN-γ and IL4-inducing abilities of the HTL epitopes [46].

### Identification and analysis of LBL (Linear B-Lymphocyte) epitopes

LBL epitopes are important for the induction of B lymphocytes to generate antibodies and has a important role in vaccine design. The ABCpred tool (http://crdd.osdd.net/raghava/abcpred/), based on recurrent neural network with a 0.51 threshold value, was utilized to estimate LBL from the selected protein sequences [47, 48]. LBLepitopes with scores > 0.8 were chosen as vaccine candidates. The AllerTOP v2.0 tool (https://www.ddg-pharmfac.net/AllerTOP/) [49], ToxinPred tool (http://crdd.osdd.net/raghava/toxinpred/) [40], and VaxiJen v2.0 tool (http://www.ddg-pharmfac.net/vaxijen/VaxiJen/VaxiJen.html) [31] were used to assess the anticipated linear B-lymphocyte epitopes’ allergenic, toxic, and antigenic profiles respectively.

### Evaluation of the human homology and epitope conservancy

We used the “epitope conservancy analysis” server (http://tools.iedb.org/conservancy/) of the IEDB resource to analyze the conservation of selected MHC class I/II epitopes. This feature demonstrates the availability of the epitope in a range of various strains. In the conservancy analysis, epitopes with 100% maximum identity were selected for vaccine construct [50]. Epitope homology with the human proteome was investigated to avoid cross reaction with human proteins or weak response due to tolerance, and non-homologous epitopes were selected. Human homology was determined using the protein BLAST module of the BLAST server (https://blast.ncbi.nlm.nih.gov/Blast.cgi) with Homo sapiens (taxid: 9606), and a threshold of 0.05.Non-homologous peptides where no hits were found below the threshold e-value were designated as epitopes [51, 52].

### Molecular docking and peptide modeling analysis

To assess the binding ability, selective MHC-I epitopes were docked with related HLA alleles. For modeling the determined CTL epitopes, we used the PEP-FOLD v3.5 tool (https://bioserv.rpbs.univ-paris-diderot.fr/services/PEP-FOLD3/). This tool utilizes the Taboo/Backtract sampling algorithm for the prediction of peptide 3D conformations with 5 − 50 residues [53]. After predicting five possible structures through this tool for any peptide sequence, the energy of each structure was determined using SWISS-PDB VIEWER, and the model with the least energy was selected for subsequent assessments [54]. The human alleles HLA-A*03:01 (PDB ID: 6O9B), HLA-B*18:01 (PDB ID: 6MT3), HLA-A*02:01 (PDB ID: 7RTD), HLA-A*24:02 (PDB ID: 7MJA), HLA-B*08:01 (PDB ID: 7NUI), HLA-B*07:02 (PDB ID: 7RZD), HLA-B*58:01 (PDB ID: 5VWJ), HLA-A*01:01 (PDB ID: 6AT9), HLA-B*44:03 (PDB ID: 4JQX), HLA-B*40:02 (PDB ID: 5IEK) were considered for MHC-I epitopes. Using the RCSB Protein Data Bank (https://www.rcsb.org/), the crystal structure of shortlisted HLA alleles was downloaded in pdb format [55]. Then, the protein preparation wizard of UCSF Chimera (version 1.11.2) was utilized to prepare proteins by removing structurally bound ligands [56]. The HADDOCK tool (https://wenmr.science.uu.nl/haddock2.4/) was used to estimate the interaction among the Alleles and CTL Epitopes [57, 58]. Molecular visualization of docking analysis was performed through Ligplot software, and images were obtained by UCSF Chimera and Microsoft PowerPoint 2019 [56, 59].

### Population coverage analysis

Variations in the HLA allele distribution and expression in regions and races around the world may affect the response to vaccines based on epitope [60]. The population coverage of the candidate vaccine was estimated by implementing the IEDB population coverage server (http://tools.iedb.org/population/). To do this, the investigation of selected HTL and CTL epitopes coupled with their relevant HLA binding alleles in both MHC (I and II) classes were performed individually and in combination [61]. In this study, our emphasis was on the global coverage of alleles and parts of different continents.

### MHC cluster analysis

The MHC gene family, as one of the most polymorphic genes in the various species' genomes, contains several thousand alleles in humans [62]. Cluster analysis of MHC alleles is used to identify two classes of MHC molecules with similar binding specificities. The MHCcluster 2.0 online tool (https://services.healthtech.dtu.dk/services/MHCcluster-2.0/) was utilized to provide highly instinctive heat maps and phylogenetic tree-based visualizations of the functional cluster between MHC variants based on the default parameters. During the MHC class I cluster analysis, the NetMHCpan-2.8 approach was utilized with an HLA-prevalent and -characterized module, while for the MHC class II cluster analysis, the relevant DRB allele modules were chosen [62, 63].

### Designing and formulation of the multi-epitope vaccine

The vaccine was constructed from the selected HTL, CTL, and LBL epitopes of HPyV6 and HPyV7 proteins. Also, an adjuvant was attached to the vaccine structure using a suitable linker [64, 65]. We used TLR4 agonist as an adjuvant because viral glycoproteins were found to recognize TLR4 agonist [66, 67]. Therefore, 50S ribosomal protein L7/L12 was included as adjuvant (NCBI ID: P9WHE3) and attached to the N-terminal of the vaccine peptides through a bifunctional linker (EAAAK). Contrastingly, the HTL, CTL, and LBL epitopes were connected using Gly-Pro-Gly-Pro-Gly (GPGPG), Ala-Ala-Tyr (AAY), and Lys-Lys (KK) linkers, respectively [64, 65]. The GPGPG linker inhibits the formation of the "junctional epitope" and aids in immune processing. The AAY linker improves epitope immunogenicity by affecting peptide stability. The KK linker improves the maintenance of independent immunogenic functions of the constructed vaccine [68, 69].

### Antigenicity, allergenicity, solubility, and physicochemical property assessment

The ProtParam tool (https://web.expasy.org/protparam/)was applied to analyze the physicochemical profiles of the constructed vaccine [70]. We also used the Vaxijen v2.0 tool (http://www.ddg-pharmfac.net/vaxijen/VaxiJen/VaxiJen.html) [31] and ANTIGENPro server (https://scratch.proteomics.ics.uci.edu/) of the Scratch protein forecast tool to predict antigenicity. ANTIGENPro demonstrated an accuracy of 76% with cross-validation tests on the combined dataset [32]. Three servers including AllergenFP v1.0 (https://www.ddg-pharmfac.net/AllergenFP/) [33], AllerTop v2.0 (https://www.ddg-pharmfac.net/AllerTOP/) [39], and AlgPred (http://crdd.osdd.net/raghava/algpred/) [71] were used to predict the allergenic profile of the vaccine construct. The SOLpro online tool (https://scratch.proteomics.ics.uci.edu/) was applied to estimate the solubility of the proposed vaccine and a prediction score ≥ 0.5, indicates the vaccine will be soluble [72]. In addition, we applied the Protein-Sol online tool (https://protein-sol.manchester.ac.uk/) to better understand the solubility [73]. By comparing the scaled solubility value (QuerySol) with the E. coli proteins’ mean solubility from the experimental dataset (PopAvrSol), solubility is predicted, and a predicted score greater than 0.45 is considered to be soluble [74]. In order to predict the number of transmembrane helices, we used the TMHMM v2.0 tool (https://services.healthtech.dtu.dk/services/TMHMM-2.0/) [34]. Also, possible signal peptides were investigated using the application of the SignalP 4.1 tool (https://services.healthtech.dtu.dk/services/SignalP-4.1/) in the ultimate designed vaccine [75].

### BLAST homology assessment

The PSI-BLAST algorithm of the NCBI Protein BLAST (BLASTp) module was used to determine the homology between the vaccine construct and the human proteome [76, 77]. Cross-checking study was performed to avoid autoimmune reactions through molecular imitation. The BLASTp search limited the results to records from H. sapiens (taxid: 9606) only. In order to be valid, the query coverage must not show more than 40% homology to the human proteome [78].

### Prediction of the secondary structure

The secondary structural configurations were identified using two servers PSIPRED v4.0 (http://bioinf.cs.ucl.ac.uk/psipred/) and Prabi (https://npsa-prabi.ibcp.fr/cgi-bin/npsa_automat.pl?page=/NPSA/npsa_gor4.html) with default parameters [79, 80]. The percentage of 2D features, i.e. α-helix, β-turns, and random coils was calculated on the vaccine construct by both servers. The precision of prediction results with the Prabi tool was reported to be a mean of 64.4% [80]. The PSIPRED server, as the most accurate predictive generator of secondary structure, displays an accuracy of 78.1% [81].

### Homology modeling and 3D structure refinement and validation

I-TASSER tool (https://seq2fun.dcmb.med.umich.edu//I-TASSER/) was used to generate the three-dimensional model of the multi-epitope vaccine. Using threading templates as templates, this server produces a 3D structure based on the amino acids sequence, and it estimates the C-score to assess the validity of the predicted models. The C-score of a model typically falls within the range of -5 to 2, with a higher C-score indicating greater confidence [82–84]. Tertiary structure vaccine model refinement was performed via the GalaxyRefine tool (https://galaxy.seoklab.org/cgi-bin/submit.cgi?type=REFINE). Various parameters including GDT-HA, rmsd, poor rotations, Molprobity, clash score, and Rama-favored are produced in the output result of five refined models [85]. After validation of the models through the ProSA-web online tool (https://prosa.services.came.sbg.ac.at/prosa.php), the estimation of the Z-score and analysis of the stereochemical quality of each protein structure were performed by assessing the residue by residue geometry and overall structure geometry [86]. In order to further analyze the Ramachandran plot, the Procheck web server (https://saves.mbi.ucla.edu/) was used to determine the overall quality of the refined 3D structure of the vaccine. The Ramachandran plot is a plot of the dihedral angles phi (ϕ) and psi (ψ) of amino acids to visualize the percentage of amino acids in the generously allowed, disallowed, most favorite, and additional allowed areas [87].

### Identification of discontinuous B-Cell epitopes

The ElliPro server (http://tools.iedb.org/ellipro/) was used to estimate conformational B-cell epitopes in the designed vaccine utilizing default parameters (minimum score: 0.5; maximum distance: 6 Å). The improved Thornton’s technique using residue clustering algorithms is the basis of the results. Prediction is done based on the neighbor residue clustering, protein form, and residual protein index (PI) [88].

### Molecular docking of the immune receptor (TLR4) and designed vaccine

The protein data bank (RCSB) at 2.4 Å resolution was used to retrieve TLR4 complexes (PDB ID: 4G8A) [55]. Heteroatoms and three chains B, C, and D were deleted in the UCSF Chimera software (version 1.11.2) [56]. Energy minimization of protein was carried out using the Swiss-PDB Viewer with the GROMOS 43B1 force field [54]. Molecular docking of vaccine-TLR4 complexes was performed in Cluspro (https://cluspro.bu.edu/login.php) online tools [89]. It's an automated, web-based program for the docking of peptide − protein or protein − protein. The server executes a series of three computational procedures in the following manner: firstly, the process of rigid body docking is carried out employing PIPER; secondly, the 1000 docked structures with the lowest energy are subjected to clustering using pairwise IRMSD as the distance metric; and finally, the forecasted complex structures positioned at the cluster centers are refined by minimizing their energy. Also, We used the balanced coefficient to obtain the best protein–protein binding results [90]. The output of this server is a short list of putative complexes ranked according to their clustering properties.

### In silico immune simulation

The immune simulation study was conducted using the C-ImmSim server (https://kraken.iac.rm.cnr.it/C-IMMSIM/index.php?page=1) to understand and investigate the immunogenicity and immune response profile. Using the position-specific scoring matrix (PSSM), this server employs real lifelike immune responses and interactions, and machine learning [91]. The time steps in the CImmSim web tool (with default parameters) were set to 1, 42, and 84, each time step is equal to 8 h and time step 1 is injection at time = 0. The time interval between two injections (a total of 3 injections) was considered 4 weeks [92].

### Molecular dynamics simulation

Molecular dynamics (MD) simulation was applied to refine the TLR-vaccine complex structures using GROMACS 2018 [93, 94]. The structures were centered in a dodecahedron box and filled with water using tip3 water model. To neutralize systems some molecules of water were randomly replaced by Cl- or Na + . After neutralization, the energy minimization was done using steepest descent algorithm. Equilibrating the systems was performed under 100 ps NVT at temperature of 298 K followed by 100 ps NPT ensembles at pressure of 1 bar. Electrostatic interactions were calculated by PME (Flores-Canales and Kurnikova, 2015) and the LINCS procedure was applied to constrain all bonds connecting hydrogen atoms. The Final MD simulation was run for 100 ns with no restraint.

In silico cloning and codon optimization of the final vaccine protein.

The online Java Codon Compatibility Tool (JCAT) web server (http://www.jcat.de/) was utilized for codon optimization and reverse translation of the ultimate vaccine protein [95]. To express the final construct in E. coli, the K12 strain was used as the host. Using this server, important parameters such as GC content and codon adaptive index (CAI) were calculated for assessment of protein expression levels. After introducing sites for BamHI and XhoI restriction enzymes within 3′ and 5′ends of the designed vaccine sequence, respectively, this sequence was transformed into the pET30 ( +) vector through the SnapGene software.

## Results

### Vaccine design

#### Retrieval and evaluation of protein sequences

The reference sequence for HPyV6 and HPyV7 large T antigen (LTAg), and viral proteins 1/2 (VP1, VP2) were obtained from UniProt Proteome database. VaxiJen v2.0 tool was utilized to determine the subjected protein sequences. TMHMM v2.0 was employed to anticipate the number of TM helices. The antigenicity of candidate proteins varies from 0.4281 to 0.4996, hence the proteins of interest have sufficient predicted antigenic properties. In addition, the AllergenFP server suggested the proteins are non-allergenic. To construct a multi-epitope-based vaccine, the large T, VP1 and VP2 were included. The quantity of transmembrane helices is zero. Table 3 displays the sequences of these proteins, their UniProt entries, and allergenicity, antigenicity, and TM helices.

**Table 3:** Details of the selected proteins and Their Selection Criteria

Species	UniProt entry	protein name	abbreviation	Antigenicity (Probable antigen)	allergenecity	no. of TM helices
HPyV6	D6QWG6	Large T antigen	LTAg	0.4928	Probable non-allergen	0
	D6QWG0	Viral Protein 1	VP1	0.4555	Probable non-allergen	0
	D6QWF3	Viral Protein 2	VP2	0.4288	Probable non-allergen	0
HPyV7	D6QWJ6	Large T antigen	LTAg	0.4996	Probable non-allergen	0
	D6QWJ5	Viral Protein 1	VP1	0.4281	Probable non-allergen	0
	D6QWJ3	Viral Protein 2	VP2	0.4323	Probable non-allergen	0

#### Identification and validation of CTL epitopes

Based on the specified selection range, 52 potential CTL epitopes of the candidate proteins were identified as non-allergenic, non-toxic, antigenic, as well as immunogenic (Table S1). The epitopes were calculated with NetCTL 1.2 using a combinatorial approach. Just ten predicted CTL epitopes were selected for peptide-based vaccine design. The list of the chosen CTL epitopes with their characteristics in the final vaccine is shown in Table 4.

**Table 4:** A brief list of CTL Epitopes to form the final structure of the vaccine

specie	epitopes	protein	C-score	Immunogenicity score	antigenicity	allergenicity	toxicity	epitope conservancy hit (maximum identity %)	human homology	interacting MHC-I alleles
HPyV6	LSHATLGNK	LTAg	1.2320	0.10205	1.5211	probable non-allergen	non-toxic	100	non-homologue	HLA-A*11:01, HLA-A*03:01, HLA-A*30:01
	FERWVSFGM	LTAg	1.3474	0.24276	1.2787	probable non-allergen	non-toxic	100	non-homologue	HLA-B*18:01, HLA-B*40:01, HLA-B*40:02
	WLLFVLEEL	VP2	1.1038	0.28696	0.7654	probable non-allergen	non-toxic	100	non-homologue	HLA-A*02:01, HLA-A*02:06
	IYKVEAILL	VP1	1.5128	0.21171	0.4174	probable non-allergen	non-toxic	100	non-homologue	HLA-A*24:02, HLA-B*35:03
HPyV7	MELTDVLLI	LTAg	1.2565	0.08008	1.3253	probable non-allergen	non-toxic	100	non-homologue	HLA-B*44:02, HLA-B*44:03, HLA-B*40:01, HLA-B*18:01, HLA-B*53:01, HLA-B*40:02, HLA-B*51:01
	TPKRRNLLF	LTAg	1.3998	0.01055	1.7138	probable non-allergen	non-toxic	100	non-homologue	HLA-B*08:01, HLA-B*07:02
	GPRIGSTTM	VP2		0.08343	0.7918	probable non-allergen	non-toxic	100	non-homologue	HLA-B*07:02
	LWLPQAWPW	VP2	1.0974	0.08973	0.5232	probable non-allergen	non-toxic	100	non-homologue	HLA-A*24:02, HLA-B*58:01, HLA-B*57:01, HLA-A*23:01, HLA-B*53:01
	DTMIVWEAY	VP1	2.0643	0.43299	0.5828	probable non-allergen	non-toxic	100	non-homologue	HLA-A*26:01, HLA-A*25:01, HLA-A*01:01, HLA-B*35:01, HLA-B*18:01, HLA-A*29:02, HLA-A*30:02, HLA-B*46:01
	TELLFAPQM	VP1	1.0319	0.05903	0.8241	probable non-allergen	non-toxic	100	non-homologue	HLA-B*18:01, HLA-B*44:02, HLA-B*40:02, HLA-B*44:03, HLA-B*40:01

#### Identification and validation of HTL epitopes

Overall, 55 potential HTL epitopes under specified selection range as non-allergenic, non-toxic, and antigenic were identified (Table S2). Evaluation of expected cytokine induction capability was done on selected HTL epitopes and based on those results 7 peptides were selected to include in the final vaccine. The list of the chosen HTL peptides with their characteristics in the final vaccine is shown in Table 5.

**Table 5:** A brief list of HTL Epitopes to form the final structure of the vaccine

specie	epitopes	protein	antigenicity	allergenicity	IFN-γ	IL4	toxicity	epitope conservancy hit (maximum identity %)	human homology	interacting MHC-II alleles
HPyV6	VAAQRRVLMLESTRQ	LTAg	0.8394	probable non-allergen	positive	inducer	non-toxic	100	non-homologue	HLA-DRB1*04:10, HLA-DRB1*04:04, HLA-DRB1*04:05, HLA-DRB1*04:08
	GLVNLVNYAVNYNRQ	VP2	0.7212	probable non-allergen	positive	inducer	non-toxic	100	non-homologue	HLA-DRB1*08:06, HLA-DRB1*01:02, HLA-DRB1*08:13, HLA-DRB1*13:04, HLA-DRB1*08:01, HLA-DRB1*04:23
	GRFFRVHFRQRRVKH	VP1	0.4931	probable non-allergen	positive	inducer	non-toxic	100	non-homologue	HLA-DRB1*11:20, HLA-DRB1*11:14, HLA-DRB1*13:23, HLA-DRB1*08:04, HLA-DRB5*01:01, HLA-DRB5*01:05
	SAGYIRAQGTPAGVE	VP1	0.8598	probable non-allergen	positive	inducer	non-toxic	100	non-homologue	HLA-DRB1*09:01
HPyV7	DWNFVADFAADMELT	LTAg	1.2109	probable non-allergen	positive	inducer	non-toxic	100	non-homologue	HLA-DRB3*01:01, HLA-DRB1*03:09, HLA-DRB1*03:05, HLA-DRB1*04:21
	FEPGGVVMYDTQNLP	VP2	0.9125	probable non-allergen	negative	inducer	non-toxic	100	non-homologue	HLA-DRB1*15:06, HLA-DRB1*04:02, HLA-DRB1*08:06, HLA-DRB1*15:02, HLA-DRB1*08:04, HLA-DRB1*08:13, HLA-DRB1*04:10, HLA-DRB1*08:01
	FFRVHCRQRRIKHPY	VP1	0.4266	probable non-allergen	negative	inducer	non-toxic	100	non-homologue	HLA-DRB5*01:05, HLA-DRB1*11:20, HLA-DRB1*11:14, HLA-DRB1*13:23

#### Identification and validation of linear B‑cell epitope (LBL)

Overall, 46 LBL epitopes from the target proteins were identified by evaluating potential toxicity, immunogenicity and antigenic characteristics (Table S3). One LBL epitope was chosen from each of the 6 protein components for usage in the ultimate vaccine. The list of the six LBL epitopes with their characteristics in the final vaccine is shown in the Table 6.

**Table 6:** A brief list of LBL Epitopes to form the final structure of the vaccine

specie	epitopes	protein	protein probability score	antigenicity	allergenicity	toxicity	human homology
HPyV6	SSEVRPPPQYGSPGWE	LTAg	0.84	1.0311	probable non-allergen	non-toxic	non-homologue
	PSKENQPSVAGIKATR	VP1	0.88	1.3222	probable non-allergen	non-toxic	non-homologue
	SNKKRRSGGYGNSATF	VP2	0.80	0.7625	probable non-allergen	non-toxic	non-homologue
HPyV7	DSKYSATPPKQKKPNP	LTAg	0.90	1.1870	probable non-allergen	non-toxic	non-homologue
	PATIPPTVEGGLGFAP	VP1	0.88	1.1122	probable non-allergen	non-toxic	non-homologue
	DQRGGFHDEGTWVSFQ	VP2	0.81	1.1021	probable non-allergen	non-toxic	non-homologue

### Vaccine evaluation

#### Evaluation of human homology and epitope conservancy

A lack of homology to normal human proteins was assessed for each of the shortlisted epitopes in both MHC classes and no homologies were identified in the human proteome, suggesting that responses against these peptides are not likely to cause response against a normal protein. Tables 4 and 5 have incorporated conservancy and human homology analyses of selected epitopes.

#### Molecular docking analyses of the CTL epitopes and HLA alleles

**Fig. 1:**
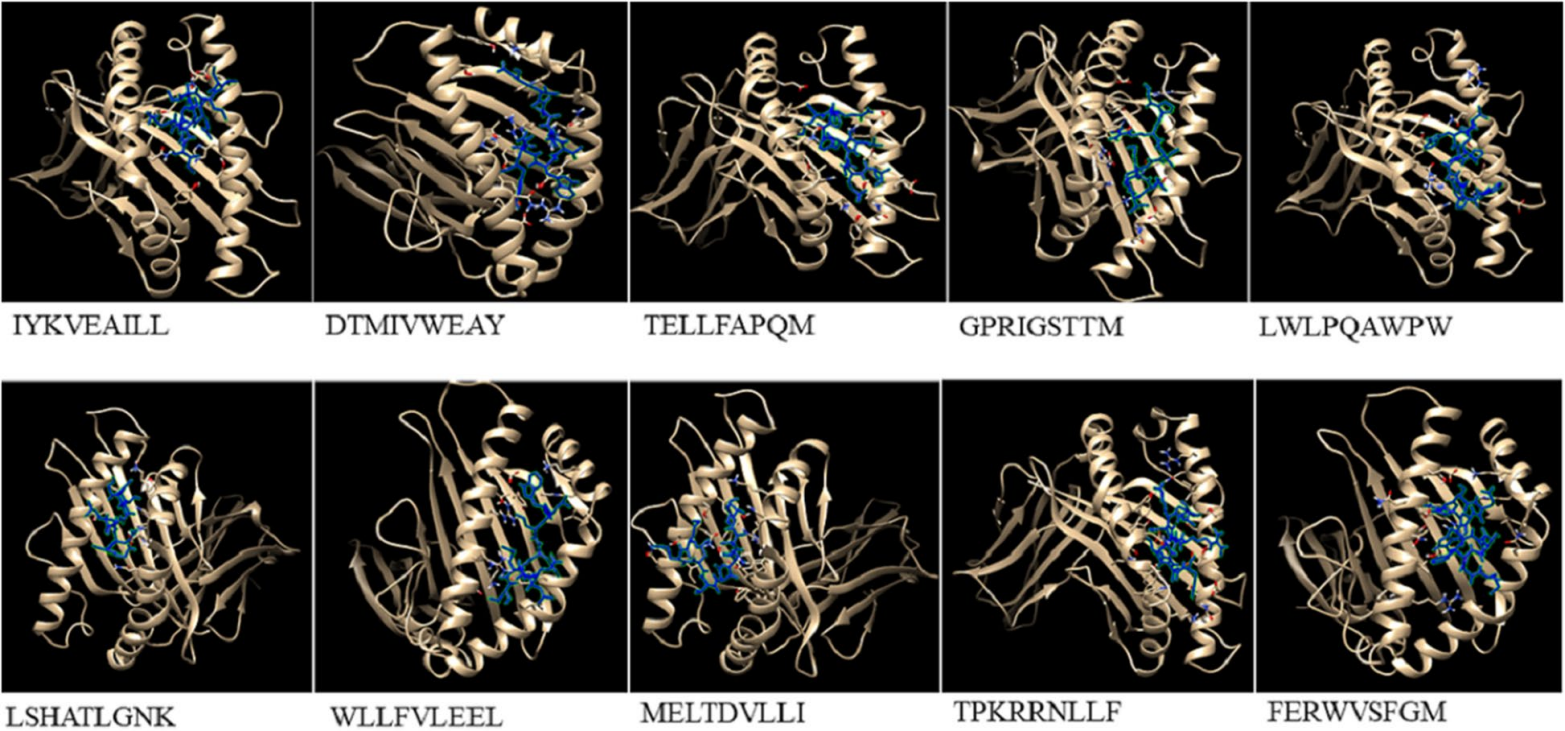
Molecular docking of the selected CTL epitopes with their respective HLA alleles as indicated in Table 5

Utilizing molecular docking simulations, we assessed CTL epitope binding to alleles of HLA. We have elected the HLA-A*03:01 allele for LSHATLGNK epitope, HLA-B*18:01 allele for FERWVSFGM epitope, HLA-A*02:01 allele for WLLFVLEEL epitope, HLA-A*24:02 allele for IYKVEAILL epitope, HLA-B*08:01 allele for TPKRRNLLF epitope, HLA-B*07:02 allele for GPRIGSTTM epitope, HLA-B*58:01 allele for LWLPQAWPW epitope, HLA-A*01:01 allele for DTMIVWEAY epitope, HLA-B*44:03 allele for MELTDVLLI epitope, and HLA-B*40:02 allele for TELLFAPQM epitope. The more negative the z-score indicates that the cluster has a high level of reliability. Based on the docking parameters, the selected CTL epitopes exhibited excellent binding interactions with the active site of HLA alleles (Fig. 1). The docking statistics are demonstrated in Table 7.

**Table 7:** Data of the molecular docking between CTL Epitopes and HLA Alleles

alleles	epitopes	HADDOCK score	Van der Waals energy	Electrostatic energy	Desolvation energy	Restraints violation energy	Buried Surface Area	RMSD from the overall lowest-energy structure	Z-Score
HLA-A*03:01	LSHATLGNK	-66.5 ± 3.3	-28.3 ± 3.5	-206.2 ± 23.6	0.2 ± 1.9	28.5 ± 24.3	1006.2 ± 77.1	1.2 ± 0.1	-1.7
HLA-B*18:01	FERWVSFGM	-101.3 ± 6.3	-45.5 ± 6.9	-112.0 ± 25.7	-39.3 ± 3.5	58.6 ± 38.3	1442.7 ± 27.8	0.5 ± 0.3	-1.6
HLA-A*02:01	WLLFVLEEL	-85.0 ± 0.4	-39.3 ± 3.4	-97.8 ± 4.0	-29.8 ± 1.6	36.2 ± 21.4	1408.9 ± 38.7	2.2 ± 0.1	-1.4
HLA-A*24:02	IYKVEAILL	-72.3 ± 2.8	-39.3 ± 5.0	-69.2 ± 14.9	-22.7 ± 1.9	35.1 ± 19.9	1143.8 ± 17.1	1.5 ± 0.1	-1.1
HLA-B*08:01	TPKRRNLLF	-99.3 ± 2.1	-43.4 ± 2.8	-220.7 ± 11.5	-12.0 ± 4.6	2.8 ± 1.2	1447.9 ± 117.0	0.6 ± 0.4	-1.6
HLA-B*07:02	GPRIGSTTM	-62.2 ± 0.8	-35.0 ± 1.6	-169.5 ± 9.4	5.9 ± 1.6	8.0 ± 7.7	1242.8 ± 24.3	0.2 ± 0.1	-1.7
HLA-B*58:01	LWLPQAWPW	-93.9 ± 1.8	-48.4 ± 2.6	-29.3 ± 8.5	-43.7 ± 2.3	40.4 ± 34.9	1296.0 ± 22.3	0.6 ± 0.1	-1.5
HLA-A*01:01	DTMIVWEAY	-108.1 ± 3.0	-52.8 ± 5.8	-181.1 ± 25.2	-19.3 ± 2.8	3.4 ± 3.5	1485.3 ± 18.1	0.4 ± 0.3	-2.3
HLA-B*44:03	MELTDVLLI	-70.2 ± 3.1	-47.4 ± 3.9	-63.0 ± 25.0	-10.5 ± 1.0	2.9 ± 1.7	1331.2 ± 24.4	0.3 ± 0.2	-1.2
HLA-B*40:02	TELLFAPQM	-82.9 ± 2.5	-46.1 ± 3.2	-87.9 ± 12.5	-20.9 ± 2.4	17.2 ± 13.6	1362.8 ± 30.6	0.4 ± 0.3	-1.5

#### Population coverage analysis

**Fig. 2:**
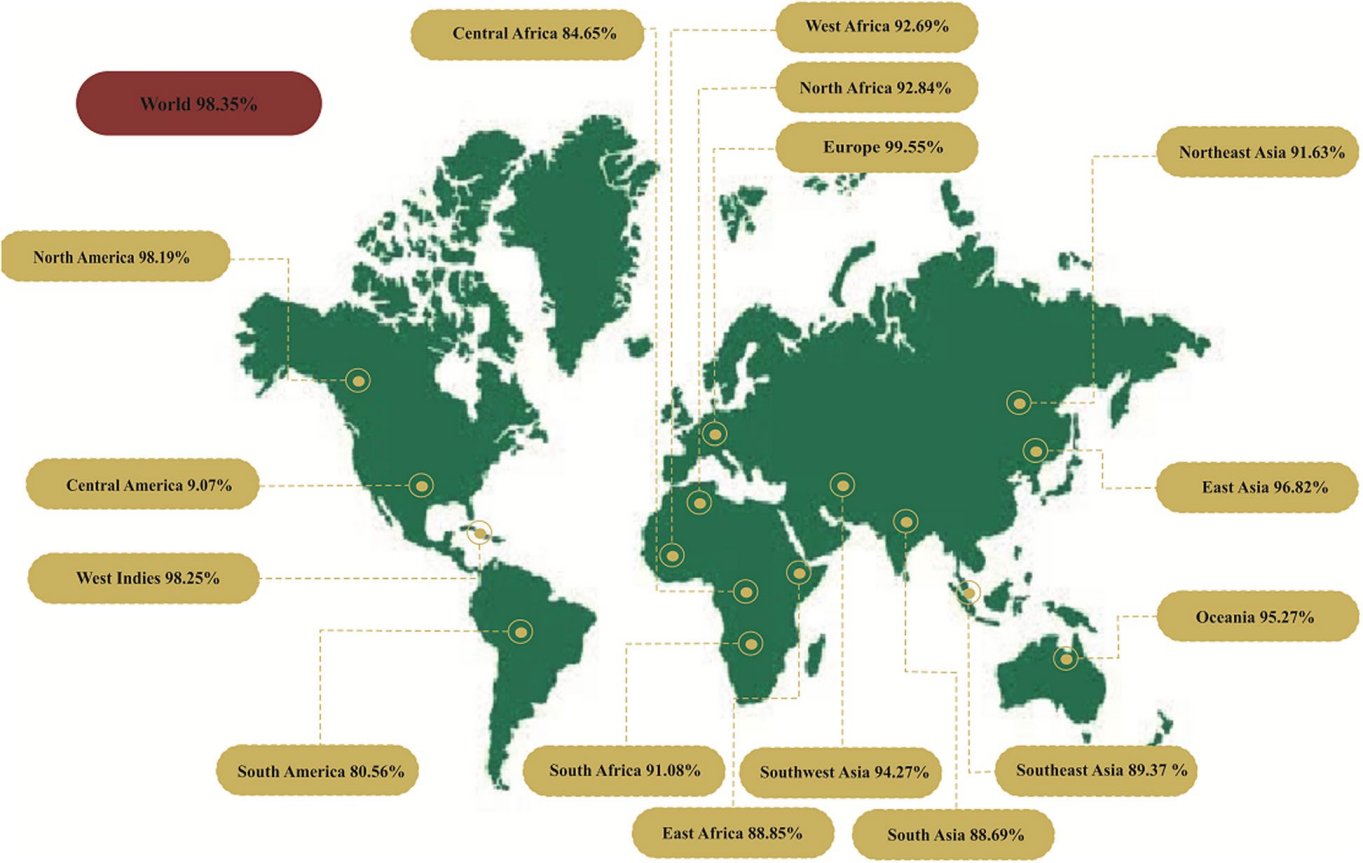
Analysis of population coverage of alleles worldwide

The analysis of MHCI and MHCII epitopes demonstrated that 97.74% of the global population is covered by MHCI epitopes, while MHCII epitopes cover 26.99% of the global population. Since a multi-epitope vaccine protein includes both MHC epitope classes, a combined estimate of their population coverage was used. Overall, 98.35% of the world's population was covered. Combining MHC class-I and class-II epitope coverage in Europe was 99.55%. followed by West Indies (98.25%), North America (98.19%), East Asia (96.82%), Oceania (95.27%), Southeast Asia (94.27%), North Africa (92.84%), West Africa (92.69%), Northeast Asia (91.63%), South Africa (91.08%), Southwest Asia (89.37%), East Africa (88.85%), South Asia (88.69%), Central Africa (84.65%), South America (80.56%) and Central America (9.07%). Comparison of population coverage between the epitopes of MHC class I/II and mixed MHC epitopes are shown in Fig. 2, Table 8, and Figures S1-S3.

**Table 8:** Analysis of MHC restriction data for worldwide population coverage

Population/Area	MHC I			MHC II			MHC I and MHC II Combined		
	Coverage(%)^a^	Average hit^b^	PC90^c^	Coverage(%)^a^	Average hit^b^	PC90^c^	Coverage(%)^a^	Average hit^b^	PC90^c^
World	97.74%	3.2	1.55	26.99%	0.34	0.14	98.35%	3.54	1.77
East Asia	96.82%	3.29	1.62	53.39%	0.66	0.21	96.82%	3.29	1.62
Northeast Asia	91.63%	2.34	1.06	33.41%	0.39	0.15	91.63%	2.34	1.06
South Asia	88.69%	1.96	0.88	24.5%	0.29	0.13	88.69%	1.96	0.88
Southeast Asia	94.27%	3.05	1.26	41.54%	0.46	0.17	94.27%	3.05	1.26
Southwest Asia	89.37%	2.16	0.94	18.19%	0.21	0.12	89.37%	2.16	0.94
Europe	99.55%	3.86	2.18	20.76%	0.28	0.13	99.55%	3.86	2.18
East Africa	88.85%	2.18	0.9	28.6%	0.39	0.14	88.85%	2.18	0.9
West Africa	92.69%	2.38	1.11	52.54%	0.66	0.21	92.69%	2.38	1.11
Central Africa	84.65%	1.94	0.65	24.28%	0.35	0.13	84.65%	1.94	0.65
North Africa	92.84%	2.38	1.11	37.7%	0.51	0.16	92.84%	2.38	1.11
South Africa	91.08%	2.16	1.04	1.79%	0.02	0.1	91.08%	2.16	1.04
West Indies	98.25%	3.4	1.77	27.52%	0.37	0.14	98.25%	3.4	1.77
North America	98.19%	3.37	1.7	24.95%	0.32	0.13	98.19%	3.37	1.7
Central America	9.07%	0.14	0.11	16.53%	0.2	0.12	9.07%	0.14	0.11
South America	80.56%	2.05	0.51	27.31%	0.35	0.14	80.56%	2.05	0.51
Oceania	95.27%	3.08	1.35	35.77%	0.4	0.16	95.27%	3.08	1.35

^a^Projected population coverage

^b^Average number of epitope hits/HLA combinations recognized by the population

^c^Minimum number of epitope hits/HLA combinations recognized by 90% of the population

#### MHC cluster analysis

**Fig. 3:**
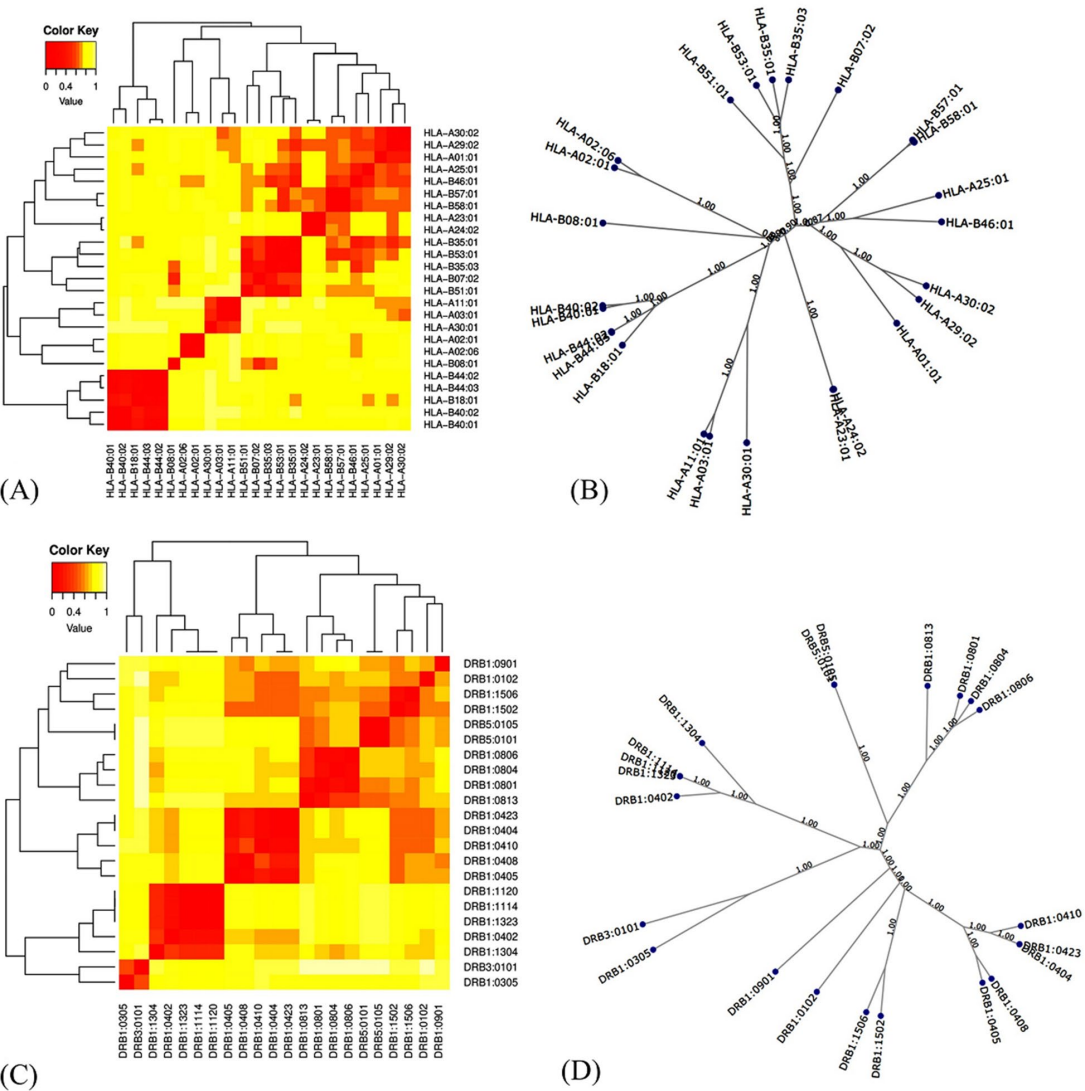
Results of the Cluster analysis for MHC I and II molecules. **A** Heat map showing the MHC-I cluster, **B** Heat map showing the MHC-II cluster, **C** detailed tree map of the MHC-I clustering analysis, **D** detailed tree map of the MHC-II clustering analysis

MHC cluster v2.0 server was exploited in order to cluster MHC classes I and II alleles that interact by selected structural protein epitopes. In this study, 25 alleles from the MHC class I, and 22 alleles from the MHC class II were analyzed. MHCI and MHC II molecules Cluster analysis is shown in Fig. 3A, C, respectively. A tree map showing the cluster analysis of MHCI and MHCII is also shown in Fig. 3B, D. The red zones on the heat map were associated with stronger interactions, whereas the yellow zones were associated with feeble interactions among clusters of both MHC molecules.

#### Formulation of the vaccine construct

**Fig. 4:**
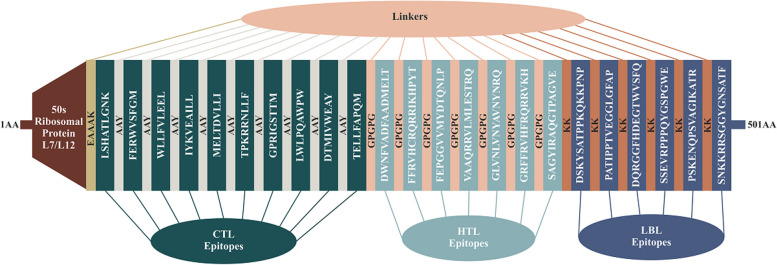
Schematic presentation of the formulated multi-epitope vaccine construct. The multi-epitope vaccine constructs included (left to right) an adjuvant and CTL, HTL, and LBL epitopes, indicated in the Brown, navy, light blue, and violet rectangular boxes, respectively

In order to formulate the vaccine construct, we assembled the most favorable CTL, HTL, and LBL epitopes using AAY, GPGPG, and KK linkers, respectively. Furthermore, 50S ribosomal protein L7/L12 adjuvant (NCBI ID: P9WHE3) was attached using EAAAK linker to the N-terminal region of the vaccine. The structure of the vaccine consists of seven epitopes of HTL, ten epitopes of CTL, and six epitopes of LBL from the target protein sequences of polyomaviruses 6 and 7. After suitable evaluation and comparison of different structures, we determined the final structure of the vaccine with 501 amino acids. The final recombinant vaccine was analyzed for subsequent evaluations (Fig. 4).

### Vaccine assessment

#### Assessment of the antigenicity, allergenicity, and physicochemical properties of the final vaccine protein

**Fig. 5:**
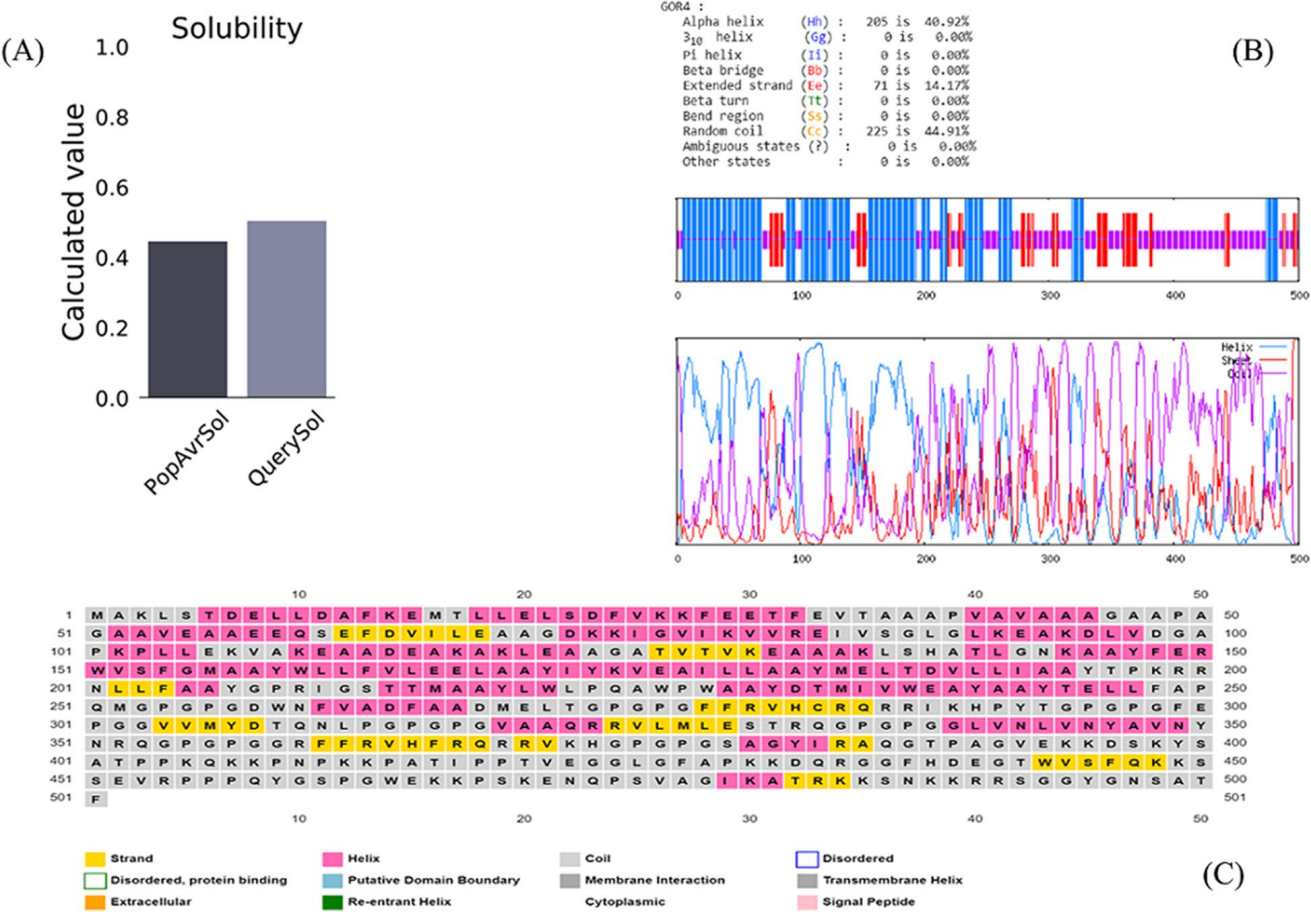
Protein-Sol server prediction of vaccine protein Solubility (**A**), Prabi server Prediction of vaccine secondary structure (**B**), PSIPRED server Prediction of vaccine secondary structure (**C**)

The physicochemical characteristics of the formulated construct were evaluated. The vaccine's chemical formula is C_2455_H_3814_N_662_O_694_S_11_. The molecular weight of the vaccine construct was estimated to be 54059.97 Da. It was calculated that the protein has a theoretical pI value of 9.41. This value represents that the protein is highly basic. The grand average of hydropathicity (GRAVY) property demonstrated the hydrophilic nature of protein, as it was-0.317. Furthermore, the instability index score was 34.33, and the score of the aliphatic index was calculated as 72.95. The subjected protein’s half-life was evaluated 30 h in mammalian reticulocytes in vitro and exceeds 20 h in yeast also approximated 10 h in E. coli in vivo. Vaccine allergenicity and antigenicity were also assessed using multiple servers. According to ANTIGENPro and Vaxijen 2.0 tools, the score of antigenicity was determined to be 0.897722 and0.6224, respectively. We also employed several tools to evaluate the solubility of the vaccine sequence. In Solpro and Protein-Sol tools, the score of solubility was estimated to be 0.777639 and 0.504, respectively. Also, the final proposed vaccine did not indicate any signal peptides and Transmembrane helices based on the prediction data (Table 9 and Fig. 5A).

**Table 9:** Allergenicity, antigenicity, and physicochemical properties of the final structure of the vaccine

characteristics	findings	remarks
Number of amino acids	501	suitable
molecular weight	54,059.97 Da	average
Theoretical pI	9.41	basic
chemical formula	C_2455_H_3814_N_662_O_694_S_11_	–-
extinction coefficient (at 280 nm in H2O)	77,810 M^−1^ cm^−1^	
estimated half-life (mammalian reticulocytes*, *in vitro)	30 h	satisfactory
estimated half-life (yeast cells, in vivo)	> 20 h	satisfactory
estimated half-life (*E. coli*, in vivo)	> 10 h	satisfactory
instability index of vaccine	34.63	Stable
aliphatic index of vaccine	72.95	thermostable
grand average of hydropathicity (GRAVY)	-0.317	hydrophilic
antigenicity	0.6455 (VaxiJen v2.0) 0.897722 (ANTIGENPro)	antigenic
allergenicity	No (AllergenFP v1.0) No (AlgPred) No (AllerTop v2.0)	non-allergen
solubility	0.766196 (SolPro) 0.504 (Protein-Sol)	soluble
TM helices	No	suitable

#### BLAST homology assessment

Homo sapiens proteins were found to be 24% homologous with the protein vaccine sequence based on sequence homology between the designed vaccine and the proteome sequence. These results confirmed that chimeric vaccine construct cannot prompt autoimmune responses in the host based on BLAST homology assessment. This study focused on the Homo sapiens species (taxid:9606).

#### Secondary structure extrapolation

The percentage of the secondary structural features of the multi-epitope vaccine was carried out using the PSIPRED and Prabi servers.

The prabi tool estimated 40.92% α-helix, 14.17% β—strand, and 44.91% random coils (Fig. 5B), whilst the PSIPRED server estimated 37.72% α—helix, 10.97% β—strand, and 51.31% random coils in the multi-epitope vaccine construct (Fig. 5C).

#### Tertiary structure modeling, refinement, and validation of the multi‑epitope vaccine

**Fig. 6:**
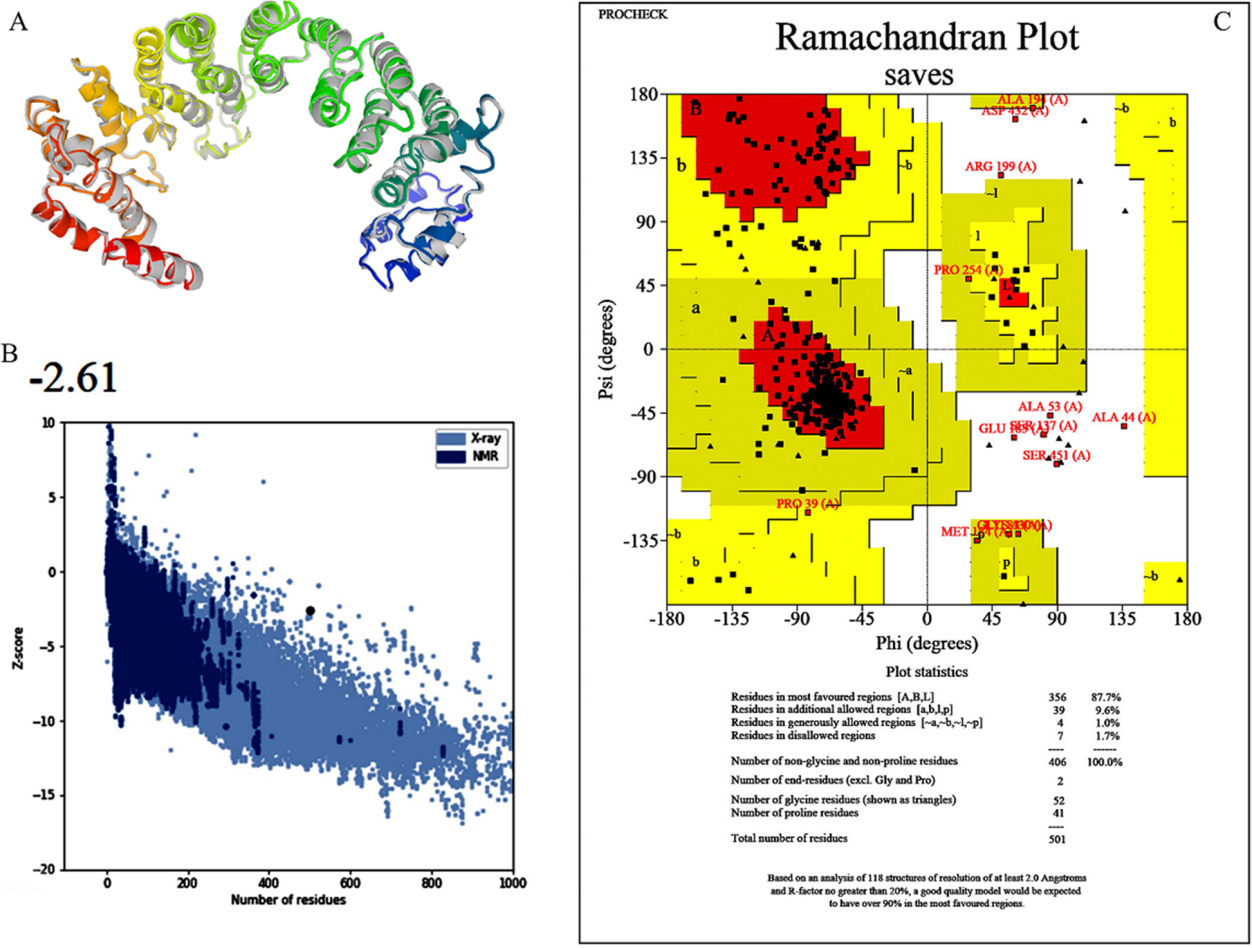
The evaluation of the ultimate 3D structure. 3D structure refined by the GalaxyRefine server (**A**), a Z-score calculated using the PROSA server for the vaccine construct (**B**), and an analysis of the vaccine construct using Ramachandran plot (**C**)

The I-TASSER online tool was utilized to make the tertiary structure of the ultimate vaccine protein. The server created 5 models for the appointed vaccine. The estimated C-score values for models 1–5 were -0.97, -1.63, -2.97, -4.44, and -3.40, respectively. The best structure with a C-score value of − 0.97 from modeling was selected for additional analysis. before refinement, Procheck and ProSA and tools were utilized to assessment this model. In the current survey, model 1 showed a z-score of -2.12 and 73.4% of the residues in the most favored regions. The GalaxyRefine tool was used to refine the 3D structure of the submitted model. This generated five refined structures for the raw model (Table 10). After refinement, all structures show the regions favored by Rama more than the submitted originally raw model. Model 3 was determined to be the best refined structure among the generated models. It displayed goodRama-favored (90.6), poor rotamers (0.8), MolProbity (2.195), clash score (14.4), rmsd (0.517), and GDT-HA (0.9037) scores. ProSA and SAVES v6.0 online tools were employed to validate the refined structure. Based on Ramachandran plot of the selected model indicated that 87.7% of amino acids in favored regions, 9.6% additional allowed, 1.0% generously allowed, and 1.7% disallowed regions were found. The Z-score value for the refined model was estimated -2.61 (Fig. 6). For further analysis, we have selected model 3 in this study.

**Table 10:** Models of vaccines refined by Galaxy Refine

Model	GDT-HA	RMSD	MolProbity	Clash score	Poor rotamers	Rama favored
Initial	1.0000	0.000	3.074	5.4	18.2	71.5
MODEL 1	0.9097	0.506	2.216	14.1	0.8	89.6
MODEL 2	0.9087	0.504	2.240	15.4	0.3	90.0
MODEL 3	0.9037	0.517	2.195	14.4	0.8	90.6
MODEL 4	0.9142	0.498	2.338	15.3	1.3	89.6
MODEL 5	0.9077	0.515	2.230	14.2	0.5	89.2

#### Screening for conformational B-Cell epitopes

**Fig. 7:**
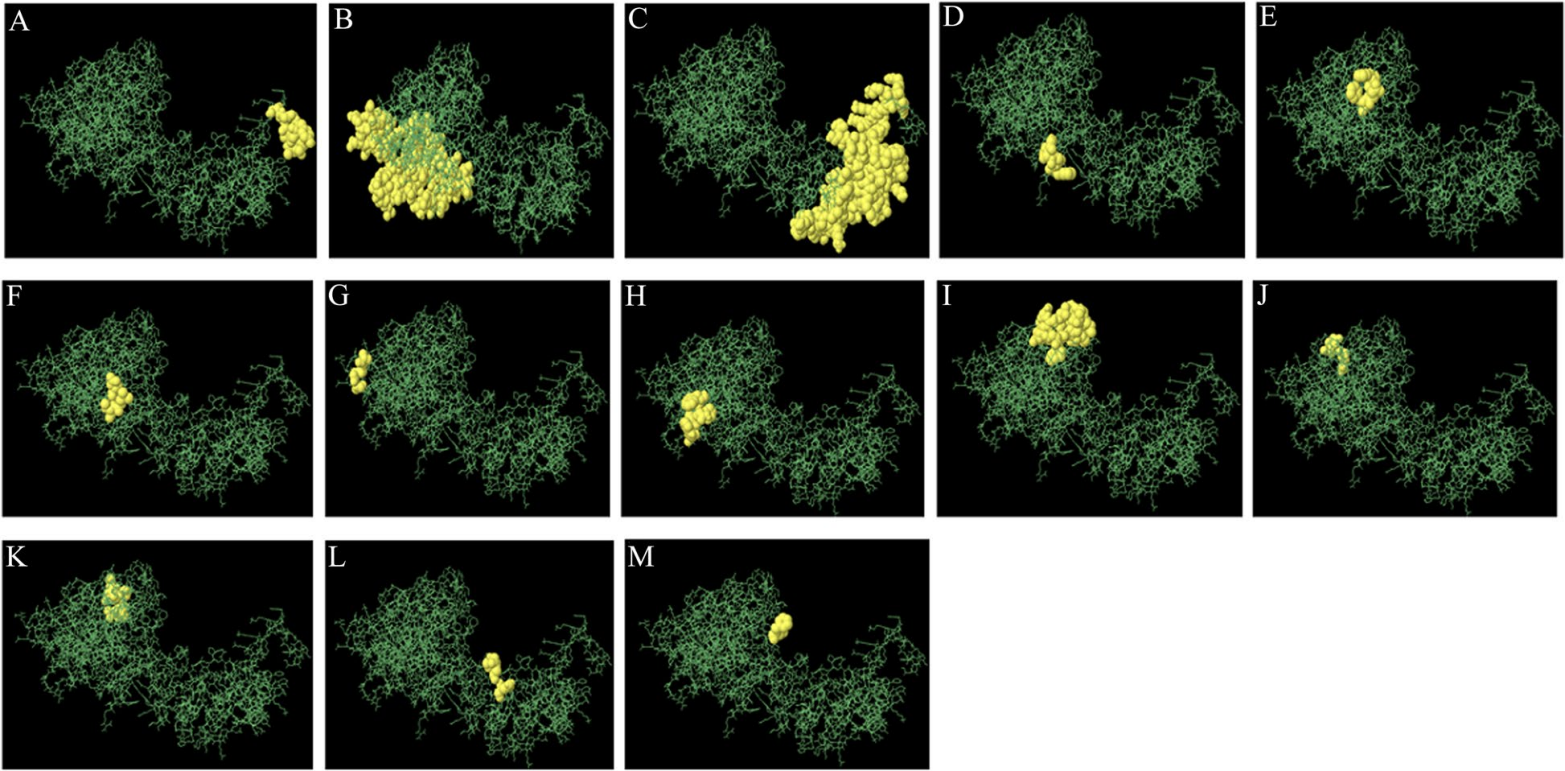
3D structure Conformational B-cell epitopes existing in the protein vaccine (**A**-**N**). Green rods and yellow domain show the protein construct and Conformational B- cell epitopes, in order

The ElliPro server identified thirteen Conformational B-cell epitopes in the vaccine construct sequence (Fig. 7). A total of 243 residues were found in these epitopes ranging in size from 3 to 98, shown in Table 11. Moreover, B—cell epitope scores ranged from 0.975 to 0.53 for the prediction of conformational B-cell epitopes.

**Table 11:** Shortlist of Conformational Epitopes of the ultimate designed vaccine

no	residues	no. of residues	score
1	B:S492, B:G493, B:G494, B:Y495, B:G496, B:N497, B:S498, B:A499, B:T500, B:F501	10	0.975
2	B:M1, B:A2, B:K3, B:L4, B:S5, B:T6, B:D7, B:E8, B:L9, B:L10, B:D11, B:A12, B:F13, B:K14, B:E15, B:M16, B:T17, B:L18, B:L19, B:E20, B:L21, B:S22, B:D23, B:F24, B:V25, B:K26, B:K27, B:F28, B:E29, B:E30, B:T31, B:F32, B:E33, B:V34, B:T35, B:A36, B:A37, B:A38, B:P39, B:V40, B:A41, B:V42, B:A43, B:A44, B:A45, B:G46, B:A47, B:P49, B:A50, B:G51, B:A52, B:A53, B:V54, B:E58, B:S61, B:E62, B:F63, B:D64, B:V65, B:I66, B:L67, B:E68, B:A69, B:A70, B:G71, B:D72, B:K73, B:K74, B:I75, B:G76, B:V77, B:I78, B:K79, B:V80, B:V81, B:R82, B:E83, B:I84, B:V85, B:S86, B:G87, B:L88	82	0.773
3	B:Q353, B:G354, B:P355, B:P357, B:G378, B:S379, B:A380, B:G381, B:R384, B:A385, B:T388, B:P389, B:G391, B:V392, B:K394, B:K395, B:D396, B:K398, B:Y399, B:S400, B:A401, B:T402, B:P403, B:K407, B:I417, B:P419, B:T420, B:V421, B:E422, B:G423, B:G424, B:L425, B:G426, B:F427, B:A428, B:P429, B:K430, B:K431, B:D432, B:Q433, B:R434, B:G435, B:G436, B:F437, B:H438, B:D439, B:E440, B:G441, B:T442, B:W443, B:V444, B:S445, B:F446, B:Q447, B:K448, B:K449, B:S450, B:S451, B:E452, B:V453, B:R454, B:P455, B:P456, B:P457, B:Q458, B:Y459, B:G460, B:S461, B:P462, B:G463, B:W464, B:E465, B:K466, B:K467, B:P468, B:S469, B:K470, B:E471, B:N472, B:Q473, B:P474, B:S475, B:V476, B:A477, B:G478, B:I479, B:K480, B:A481, B:T482, B:R483, B:K484, B:K485, B:S486, B:N487, B:K488, B:K489, B:R490, B:R491	98	0.737
4	B:G275, B:P276, B:G277, B:F278	4	0.696
5	B:F261, B:D264, B:F265	3	0.639
6	B:D268, B:L271, B:T272	3	0.632
7	B:A122, B:A123, B:G124, B:A125	4	0.582
8	B:W221, B:P223, B:Q224, B:A225, B:W228	5	0.568
9	B:L186, B:R199, B:R200, B:N201, B:L202, B:L203, B:Y207, B:R210, B:P250, B:Q251, B:M252, B:G253, B:P254, B:G255, B:P256, B:G257, B:D258, B:W259	18	0.563
10	B:K107, B:V108, B:E111	3	0.561
11	B:A100, B:P101, B:K102, B:P103, B:L104, B:L105	6	0.555
12	B:P337, B:G338, B:G339, B:N342	4	0.545
13	B:P297, B:G298, B:F299	3	0.53

#### Molecular docking of the vaccine protein and TLR complex

**Fig. 8:**
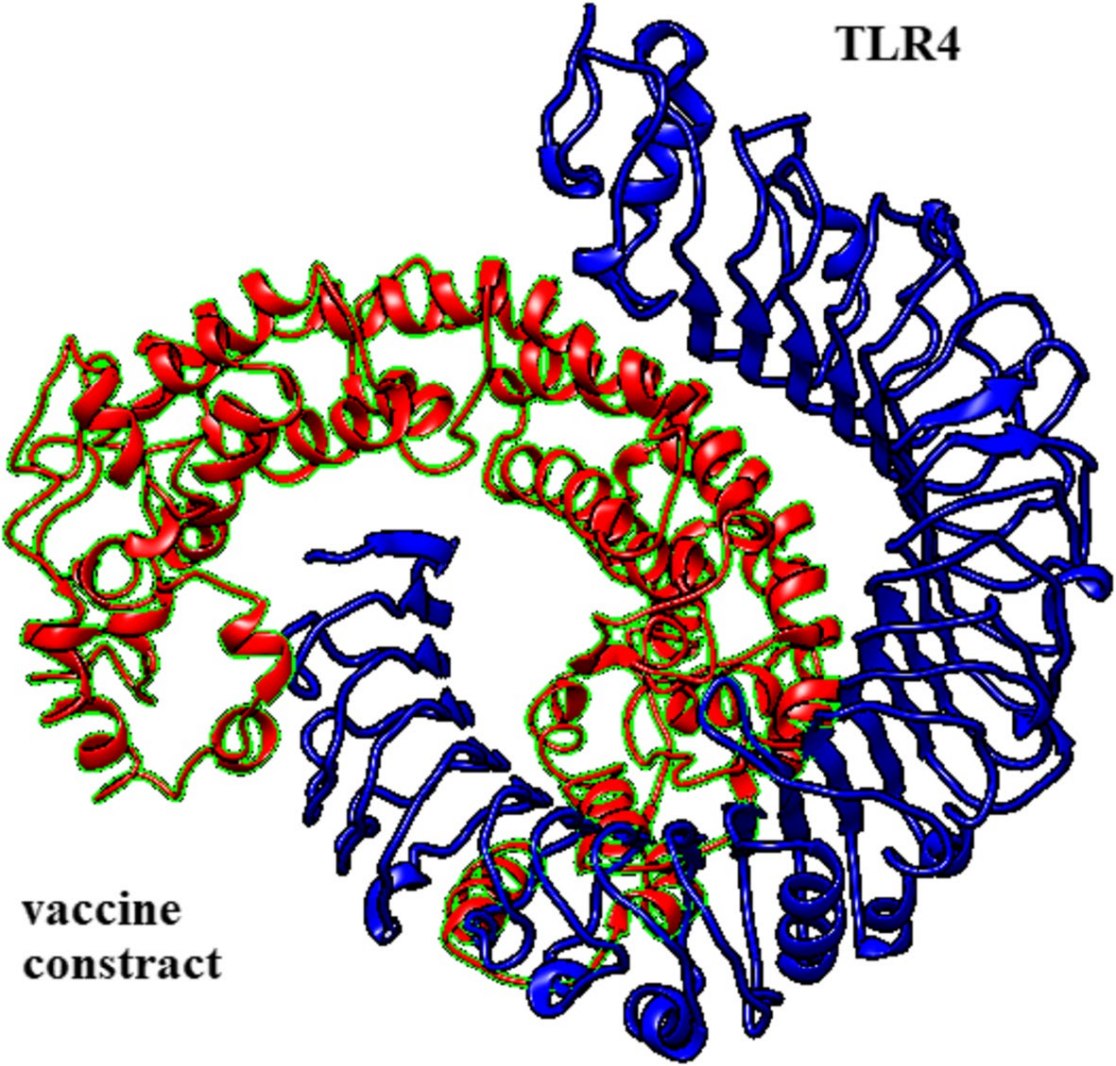
Three-dimensional representation of molecular docking of the vaccine construct and TLR complex

**Fig. 9:**
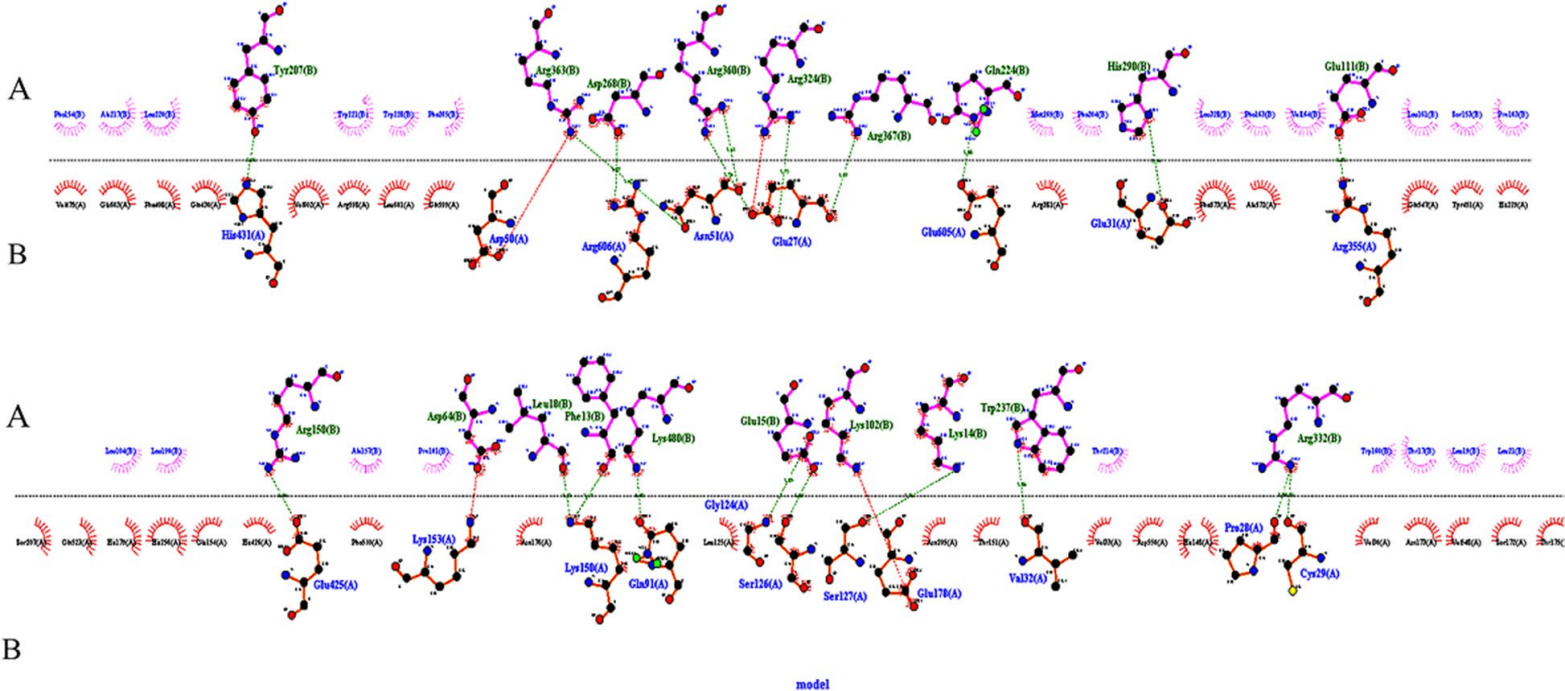
Schematic of the interaction between TLR4 and the vaccine construct. **A** Amino acids involved in hydrogen bonding from Chain A of TLR4 (**B**) Amino acids of vaccine construct

Immune cells and vaccine constructs must interact in order to produce a stable and efficient immune response. Molecular Docking of the designed vaccine with TLR4 was carried out by the ClusPro 2.0 server. In the current study, the program produced 30various clusters and ranked them by energy level. There were -1414.0, -1406.2, -1372.0, -1350.9, -1341.0, -1339.5, -1327.2, and -1321.0 kcal/mol of energy in the eight top clusters. The best group with the minimum energy of -1414.0 kcal/mol was selected. The Chimera 1.15rc program was applied to visualize the docked complex (Fig. 8). Using the LigPlot v1.4.5 software, we have generated a map with the hydrophobic interactions and hydrogen bonds between the protein vaccine and TLR4 (Fig. 9). The vaccine and chain A of TLR4 formed 20 hydrogen bonds. These hydrogen bonds are formed by amino acids along with their lengths, as shown in Table 12.

**Table 12:** Hydrogen bonding interactions between TLR4 and vaccine amino acids

TLR4 (chain A)	Vaccine	Bond length (Å)
Tyr207	His431	3.02
Arg363	Asn51	2.67
Asp268	Arg606	2.87
Arg360	Asn51	2.62
Arg360	Glu27	2.76
Arg324	Glu27	2.73
Arg367	Glu27	2.87
Gln224	Glu605	2.88
His290	Glu31	3.14
Glu111	Arg355	2.72
Arg150	Glu425	2.74
Leu18	Lys150	2.53
Phe13	Lys150	2.67
Lsy480	Gln91	2.50
Glu15	Gly124	2.89
Glu15	Ser126	2.85
Lys14	Ser127	2.76
Trp237	Val32	2.86
Arg332	Pro28	2.86
Arg332	Cys29	2.81

### Vaccine immune simulation

#### In silico immune simulation

**Fig. 10:**
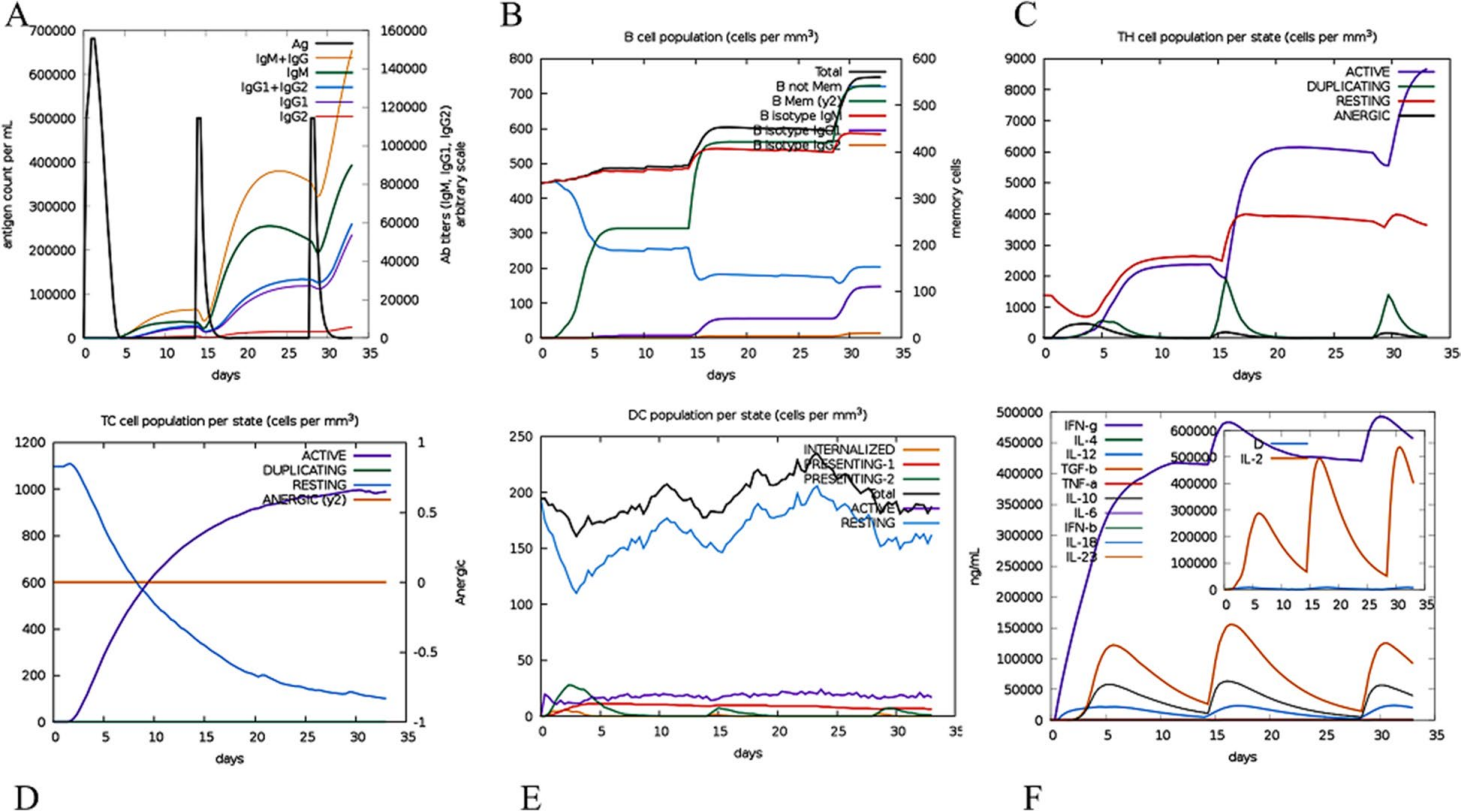
Results of the in silico immune simulation using the C-ImmSim server for the designed vaccine. **A** the generation of immune complex and immunoglobulin as a result of response to designed vaccine injections, **B** B lymphocyte total count after the three injections, **C** growth of CD4 T-helper lymphocytes after the three injections including active, duplicating, resting, anergic, **D** Increasing the number of cytotoxic CD8 lymphocytes after injection of the designed vaccine, **E** Proliferation of dendritic cells after immunization, **F** Stimulation of cytokines and interleukins after vaccine administration

Immune simulator C-ImmSim was employed to provide simulations of the immune responses associated with the final chimeric vaccine construct. The secondary and tertiary responses were clearly indicated by the anticipated elevated levels of IgM + IgG, IgM, IgG1 + IgG2, and IgG1 antibodies, subsequently followed by a reduction in antigen concentration (Fig. 10A). Results indicated a variety of long-lasting B-cell isotopes. B-cell isotype switching and memory formation may be involved in this process (Fig. 10B). In addition, T helper (helper) and TC (cytotoxic) cells are showing a clear increase with memory growth (Fig. 10C and D). There is also a clear increase in IFN-γ production and the growth of dendritic cells after immunization (Fig. 10E and F). These data represent that After successive exposures to the target antigen, robust and significant secondary immune response, antigen clearance enhancement, and production of vigorous immune memory occur.

#### Evaluation of MD simulations

**Fig. 11:**
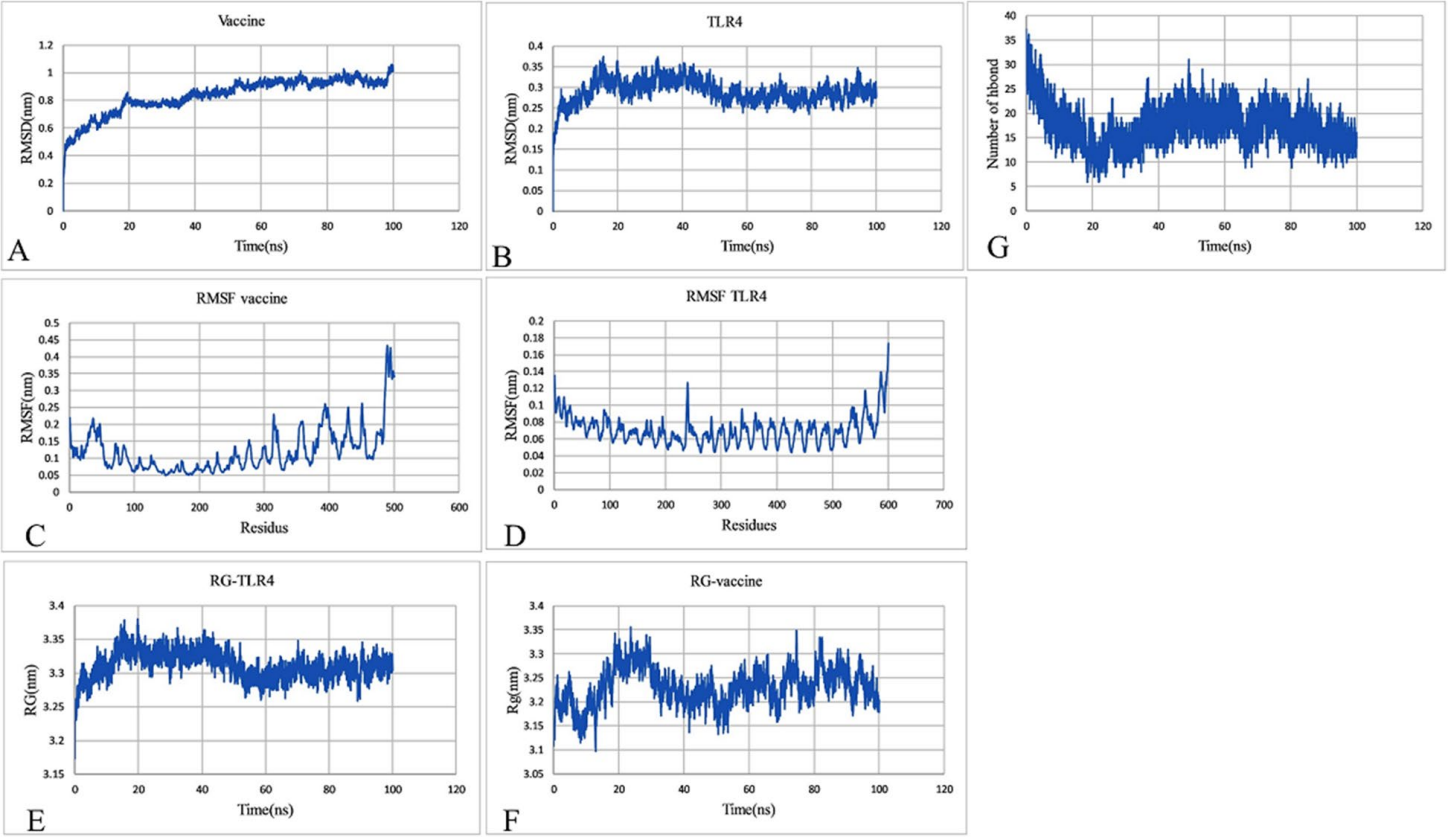
Molecular dynamics simulation of the TLR4 complex and vaccine construct. **A** RMSD plot of the vaccine construct, **B** RMSD plot of the TLR4, **C** RMSF plot of the vaccine, **D** RMSF plot of the TLR4, **E** and **F** radius of gyration of the vaccine-TLR4 complex, and **G** hydrogen bond analysis from the simulation system

The global structural stability of proteins was evaluated using Root Mean Square deviation (RMSD) of the backbone atoms. This plot shows how much the protein conformation has changed during MD simulation from initial structure. The TLR showed RMSD value in the range of 0.25 to 0.35 nm. The RMSD after 20 ns reached stability. Furthermore, the RMSD of vaccine was plotted and was in the range of 0.45 to 1. The root-mean-square fluctuation (RMSF) indicates the fluctuation of protein residues over time from a reference position during simulation. In current simulations, no unusual fluctuation was observed in protein structure. The compactness of TLR and vaccine was evaluated using Radius of gyration (Rg) plot. The Rg of TLR was in the range of 3.25 to 3.35. The Rg of vaccine was in the range of 0.3 to 0.35. A stability in compactness of each protein was observed. The hydrogen bond between receptor and peptide and was calculated. About 15 H bonds were formed between TLR and vaccine (Fig. 11).

#### Codon optimization and in silico cloning of the designed vaccine

**Fig. 12:**
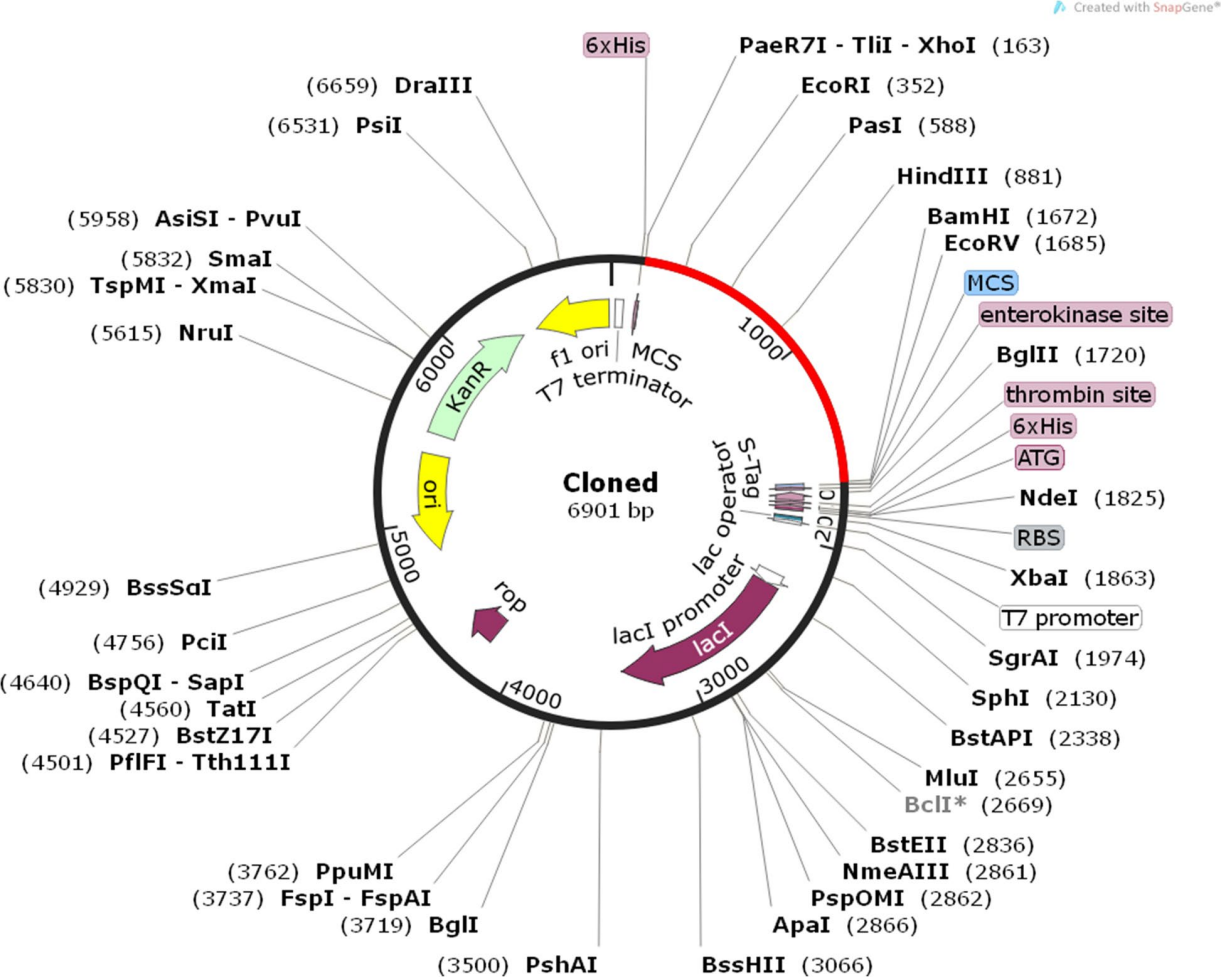
The map of the in silico cloning of the vaccine construct into the pET30a ( +) vector using SnapGene software. The black segment indicates the backbone of the vector and the red segment shows the vaccine construct. This vaccine construct contains restriction sites for XhoI and BamHI restriction enzymes at the 5′ and 3′ ends, respectively

Codon optimization is used to ameliorate gene expression and translation precision of the recombinant protein by adapting to the target host's codon bias. Reverse translation of the predicted vaccine was performed to achieve maximum expression in *Escherichia coli* strain K12 by Jcat server. After codon optimization, CAI score and GC content in improved protein sequence were estimated at 1.0 and 52.96, respectively. The data demonstrate that the improved protein sequence could be expressed sustainably in the *E. coli* system. Finally, the improved sequence was successfully integrated into the pET30a ( +) vector by SnapGene program (Fig. 12).

## Discussion

Conventional techniques for vaccine development involve the use of whole organisms, which can lead to undesired exposure to antigens and may trigger allergic responses. To prevent allergic responses, peptide-based vaccines that included short peptide fragments derived from immunogenic proteins have been used to produce strong and targeted immune reactions. Rabies, rubella, yellow fever, smallpox, hepatitis A/B, chickenpox, polio, influenza, Human Papillomavirus, and Japanese encephalitis are some of the infectious diseases which vaccines are highly effective against [96, 97]. The development of vaccines involves complex, time-consuming, and expensive in vitro and in vivo assays to ensure vaccine effectiveness [98]. Current advances in immunoinformatics and computational biology allow the design of effective vaccines in silico and reduce the number of in vitro experiments [99]. Using an in vitro study, an experimentally validated multiepitope vaccine was designed against Clostridium perfringens [100]. With this method, a wide range of vaccine candidates can be identified without the requirement of cultivating pathogenic organisms [98].

Human polyomavirus 6 (HPyV6) and HPyV7 are polyomaviruses species initially discovered in the skin of healthy people [101]. The role of HPyV6 and 7 proteins in binding and inactivating p53 has been documented, suggesting its oncogenic role [102]. The incidence of malignant skin tumors has increased over recent decades, chiefly as a result of alterable exposures.The World Health Organization reported that about 8500 new cases of skin tumors are diagnosed every day in the U.S. [7, 8]. Several studies have shown the prevalence of HPyV6- and 7 in primary cutaneous malignancies, including actinic keratosis, basal cell carcinoma, bone marrow transplantation, neuroendocrine, and lymphoid skin cancers [7].

Therefore, in the present study, the POLY capsid protein VP1, POLY minor capsid protein VP2, and POLY large T antigen from HPyV6 and HPyV7 were examined as candidate antigens for epitope identification.The allergenicity, toxicity, and antigenicity of the identified epitopes were assessed. There are a number of factors to consider when making peptide-based vaccines, including the intrinsic properties of the selected epitopes, adjuvant, and linker, and their arrangement and location within the protein. Based on the findings from the studies conducted by Olugbenga et al. [103], Mahnoor Majid et al. [104], and Sami et al. [99], we used KK, GPGPG, and AAY linkers to fuse LBL, HTL, and CTL epitopes, respectively. Epitope presentation is promoted with AAY and GPGPG linkers, while junctional epitopes are reduced with these linkers [105, 106]. The KK linker, a bi-lysine basic linker, preserved the immunogenic properties of B cell epitopes while keeping the pH near physiological levels [107, 108].

Compared with live attenuated vaccines, computational vaccines have relatively low immunogenicity. In order to address this problem, adjuvants are routinely employed. Hence, adjuvants have been widely used to increase vaccine effectiveness. Adjuvants generally function by activating innate immune cells through pathogen associated molecular pattern receptors. Adjuvants can also improve vaccines by stabilizing the epitope structure of the vaccine antigen, creating a suitable source for the gradual release of the antigen, better presenting the antigen to the antigen-presenting cells (APC), increasing the absorbing molecules of these cells at the site of the vaccine, and the proper binding of the antigen to these cells improves the vaccine performance [109]. The 50S ribosomal protein L7/L12 (Locus RL7_MYCTU) from Mycobacterium tuberculosis is a TLR4 agonist [110]. Thus, in order to enhance the immunogenicity of vaccine, we used it as an adjuvant. EAAAK, an empirical α-helical linker, reduces the connection with other protein regions while providing rigidity and improving chimeric protein durability [111]. Multiple servers determined that the construct vaccine was non-allergenic and highly antigenic, demonstrating that triggers robust immune responses without inducing unwanted allergies.

The final vaccine had a theoretical pI of 8.3, indicating its alkaline nature. Furthermore, the vaccine construct exhibited an average molecular weight of 54.05 KDa, indicating its favorable antigenic characteristics [112]. According to standards, proteins with a molecular weight below 110 kDa are deemed appropriate vaccine candidates [113]. The instability index of the vaccine was measured as 34.63, Values less than 40 are considered as a stable protein in biological environments [114]. The constructed vaccine has an indicated average half-life of above 20, 10, and 30 h in yeast cells (in vivo), E. coli (in vivo), and mammalian reticulocytes (in vitro), respectively. On the basis of previous findings, these half-life results are acceptable [99, 115]. The aliphatic index was 72.95, indicating the constructed vaccine would be thermostable at natural human body temperature [116]. The GRAVY value of the protein was -0.317, indicating the hydrophilic nature of the vaccine. Vaccine formulation and purification are made easier by the strong affinity for water molecules [117, 118].

After making the 3D model of the vaccine, the refinement system is employed to enhance its quality, both in terms of global and local structures. Validation of the model is necessary to accurately compare the unrefined structure with the refined structure. The Ramachandran plot indicated that 73.4% of the amino acid residues in the unrefined structure were detected in the desired region, while 87.7% of the amino acid residues in the refined structure were placed in the desired region, demonstrating improvement in the refined structure. Assessment of the immune response induced by an antigen is one of the primary characteristics in the validation of an introduced vaccine [119]. Molecular analyzes were employed to investigate the molecular connection between the formulated vaccine and TLR4, and suitable interactions were detected with a strong affinity score of -1414.0 kcal/mol. This relationship of the engineered vaccine with TLR-4 demonstrated that the recombinant protein vaccine has the capacity to stimulate an innate and adaptive immune response. To investigate the stability and dynamic efficiency of the vaccine/TLR4 complex, MD simulation was performed and the RMSD diagram confirmed the stable binding of this compound.

An appropriate host is required for the expression of recombinant protein. *E. coli* expression systems is the most common host for expressing recombinant proteins [120, 121]. To enable the recombinant vaccine to be expressed at high levels in *E. coli* (K12 strain) codon optimization was performed. An analysis of the designed vaccine indicated a CAI score of 1.0 and GC content of 52.96. CAI values ​​of more than 0.8 and GC content of 30–70% have been reported to favor high expression in the E. coli host [122, 123].

## Conclusion

Human polyomaviruses (HPyVs) infect a wide range of tissues such as skin, kidney and respiratory tract and often lead to persistent and asymptomatic infection, while these infections can lead to cancer. Currently, no significant therapeutic vaccine is available for HPyV. In this study, immunoinformatics techniques were applied to identify and refine candidate vaccines against HPyV. The highly immunogenic T and B cell epitopes were identified and used for vaccine design. The proposed vaccine is projected to produce robust immune reactions, including cytokines, and interferons. The binding analysis confirmed the vaccine binding to the immune receptor TLR4 that was dynamically stable. Although experimental trials in appropriate animal models is necessary to test the potency of the engineered vaccine, analysis using different bioinformatics tools indicated the high immunogenicity and preventive potential of the developed vaccine.

### Supplementary Information

**Additional file 1.**

## Data Availability

Datasets used in the experiments are listed as follows: (1) NCBI: https://www.ncbi.nlm.nih.gov/ (2) UniProt database: https://www.uniprot.org/ (3) VaxiJen server: http://www.ddg-pharmfac.net/vaxijen/VaxiJen/VaxiJen.html (4) AllergenFP v1.0 server: https://ddg-pharmfac.net/AllergenFP/ (5) TMHMM v2.0 tool: https://services.healthtech.dtu.dk/services/TMHMM-2.0/ (6) NetCTL 1.2 server: https://services.healthtech.dtu.dk/services/NetCTL-1.2/ (7) IEBD server: http://tools.iedb.org/mhci/ (8) AllerTOP v2.0 server: https://www.ddg-pharmfac.net/AllerTOP/ (9) ToxinPred server: http://crdd.osdd.net/raghava/toxinpred/ (10) Class I Immunogenicity: http://tools.iedb.org/immunogenicity/ (11) IEDB resource http://tools.iedb.org/mhcii/ (12) IFNepitope tool: http://crdd.osdd.net/raghava/ifnepitope/design.php (13) IL4pred tool: https://webs.iiitd.edu.in/raghava/il4pred/ (14) ABCpred tool: http://crdd.osdd.net/raghava/abcpred/ (15) epitope conservancy analysis: http://tools.iedb.org/conservancy/ (16) BLAST server: https://blast.ncbi.nlm.nih.gov/Blast.cgi (17) PEP-FOLD v3.5 tool: https://bioserv.rpbs.univ-paris-diderot.fr/services/PEP-FOLD3/ (18) HADDOCK tool: https://wenmr.science.uu.nl/haddock2.4/ (19) RCSB Protein Data Bank: https://www.rcsb.org/ (20) IEDB population coverage server: http://tools.iedb.org/population/ (21) MHCcluster 2.0 tool: https://services.healthtech.dtu.dk/services/MHCcluster-2.0/ (22) ExPASy ProtParam tool: https://web.expasy.org/protparam/ (23) ANTIGENPro server: https://scratch.proteomics.ics.uci.edu/ (24) AlgPred: http://crdd.osdd.net/raghava/algpred/ (25) SOLpro tool: https://scratch.proteomics.ics.uci.edu/ (26) SignalP 4.1 tool: https://services.healthtech.dtu.dk/services/SignalP-4.1/ (27) PSIPRED v4.0: http://bioinf.cs.ucl.ac.uk/psipred/ (28) Prabi: https://npsa-prabi.ibcp.fr/cgi-bin/npsa_automat.pl?page=/NPSA/npsa_gor4.html (29) I-TASSER tool: https://seq2fun.dcmb.med.umich.edu//I-TASSER/ (30) GalaxyRefine tool: https://galaxy.seoklab.org/cgi-bin/submit.cgi?type=REFINE (31) ProSA-web: https://prosa.services.came.sbg.ac.at/prosa.php (32) Procheck server: https://saves.mbi.ucla.edu/ (33) ElliPro server: http://tools.iedb.org/ellipro/ (34) ClusPro: https://cluspro.bu.edu/login.php (35) JCat: http://www.jcat.de/ (36) C-ImmSim server: https://kraken.iac.rm.cnr.it/C-IMMSIM/index.php?page=1 All data generated or analyzed during this study are included in this published article [and its supplementary information files].

## Abbreviations

HPyV6: Human polyomaviruses 6
HPyV7: Human polyomaviruses 7
CTLs: Cytotoxic T-lymphocytes
HTLs: Helper T cells
LBL: Linear B-Lymphocyte
LTAg: Large T antigen
VP1: POLY capsid protein
VP2: POLY minor capsid protein
MHC I: Major histocompatibility complex (MHC) class I
MHC II: Major histocompatibility complex (MHC) class II
RMSD: Root Mean Square Deviation
RMSF: Root Mean Square Fluctuation
Rg: Radius of gyration
TLR4: Toll like receptor 4
CAI: Codon Adaptation Index
GRAVY: Grand average of hydropathicity
pI: Isoelectric point
MD: Molecular dynamics
