# Supplementary material for: Development of a novel multi‑epitope vaccine against the pathogenic human polyomavirus V6/7 using reverse vaccinology
Source: BMC Infect Dis. 2024 Feb 9;24:177. doi: 10.1186/s12879-024-09046-0 (PMC10854057; doi:10.1186/s12879-024-09046-0)
Supplement: Supplementary file 1 — Additional file 1. [file 12879_2024_9046_MOESM1_ESM.docx]

Table S1: Analysis of HPyV structural proteins to predict potential CTL epitopes.

| species | Epitopes | Protein | C-score | Immunogenecity | Antigenecity | Allergenecity | Toxicity |
| --- | --- | --- | --- | --- | --- | --- | --- |
| HPyV6 | LLGYYIEFA | LTAg | 1.0108 | positive | 0.8134 | Non-Allergen | Non-Toxin |
|  | QLLGAVAWL | LTAg | 1.2090 | Positive | 0.4254 | Non-Allergen | Non-Toxin |
|  | FERWVSFGM | LTAg | 1.5602 | Positive | 1.2787 | Non-Allergen | Non-Toxin |
|  | TLAAAILDL | LTAg | 1.1425 | Positive | 0.6797 | Non-Allergen | Non-Toxin |
|  | MACWGNLPL | LTAg | 0.9466 | Positive | 0.6802 | Non-Allergen | Non-Toxin |
|  | ALLGYYIEF | LTAg | 1.1702 | Positive | 0.8434 | Non-Allergen | Non-Toxin |
|  | YGSPGWEQW | LTAg | 1.7179 | Positive | 1.0541 | Non-Allergen | Non-Toxin |
|  | TQLLGAVAW | LTAg | 1.0863 | positive | 0.4280 | Non-Allergen | Non-Toxin |
|  | LSHATLGNK | LTAg | 1.2320 | positive | 1.5211 | Non-Allergen | Non-Toxin |
|  | AILDLVGGV | LTAg | 1.0444 | Positive | 0.6130 | Non-Allergen | Non-Toxin |
|  | TPDKINFEL | LTAg | 1.3690 | Positive | 1.1511 | Non-Allergen | Non-Toxin |
|  | CTISFLLIK | LTAg | 1.1755 | Positive | 0.6491 | Non-Allergen | Non-Toxin |
|  | KPNPAPNDF | LTAg | 1.2513 | Positive | 1.2493 | Non-Allergen | Non-Toxin |
|  | PRRHRVSAM | LTAg | 1.2113 | Positive | 0.6859 | Non-Allergen | Non-Toxin |
|  | EEVRELMDL | LTAg | 1.4415 | Positive | 0.4086 | Non-Allergen | Non-Toxin |
|  | IYKVEAILL | VP1 | 1.5128 | positive | 0.4174 | Non-Allergen | Non-Toxin |
|  | RKQVTAANF | VP1 | 0.9676 | positive | 1.0556 | Non-Allergen | Non-Toxin |
|  | QSRGAPYTF | VP1 | 1.3706 | Positive | 0.7897 | Non-Allergen | Non-Toxin |
|  | SLCYTLAVV | VP1 | 0.9361 | positive | 1.2526 | Non-Allergen | Non-Toxin |
|  | NLVNYAVNY | VP2 | 1.2064 | Positive | 0.4152 | Non-Allergen | Non-Toxin |
|  | WLLFVLEEL | VP2 | 1.1038 | Positive | 0.7654 | Non-Allergen | Non-Toxin |
|  | LSRVRETVI | VP2 | 1.1826 | Positive | 1.1826 | Non-Allergen | Non-Toxin |
|  | SEATAALAV | VP2 | 1.2646 | Positive | 1.2646 | Non-Allergen | Non-Toxin |
|  | LEIAGGLGA | VP2 | 0.9401 | Positive | 0.9401 | Non-Allergen | Non-Toxin |
|  | VAGVGGVIY | VP2 | 1.0376 | Positive | 0.4656 | Non-Allergen | Non-Toxin |
| HPyV7 | LLPQFTIKI | LTAg | 1.1842 | positive | 0.9099 | Non-Allergen | Non-Toxin |
|  | QLIGAVAWL | LTAg | 1.1626 | positive | 0.4642 | Non-Allergen | Non-Toxin |
|  | LLGGVALNV | LTAg | 0.9494 | Positive | 0.7358 | Non-Allergen | Non-Toxin |
|  | CLLPQFTIK | LTAg | 1.4616 | Positive | 1.2585 | Non-Allergen | Non-Toxin |
|  | LSHATLGNK | LTAg | 1.2378 | Positive | 1.5211 | Non-Allergen | Non-Toxin |
|  | KLLGRDEVK | LTAg | 0.9977 | Positive | 0.5255 | Non-Allergen | Non-Toxin |
|  | TPKRRNLLF | LTAg | 1.9166 | Positive | 1.7138 | Non-Allergen | Non-Toxin |
|  | MACWGNLPL | LTAg | 0.9162 | Positive | 0.6802 | Non-Allergen | Non-Toxin |
|  | VLAARRVLM | LTAg | 1.4343 | positive | 0.6930 | Non-Allergen | Non-Toxin |
|  | FEQWVSYGM | LTAg | 1.5447 | positive | 0.5934 | Non-Allergen | Non-Toxin |
|  | MELTDVLLI | LTAg | 1.2565 | Positive | 1.3253 | Non-Allergen | Non-Toxin |
|  | ADFAADMEL | LTAg | 1.1207 | Positive | 0.748 | Non-Allergen | Non-Toxin |
|  | KSLDANPIL | LTAg | 1.0001 | Positive | 0.897 | Non-Allergen | Non-Toxin |
|  | DTMIVWEAY | VP1 | 2.0643 | Positive | 0.5828 | Non-Allergen | Non-Toxin |
|  | GRFFRVHCR | VP1 | 1.3653 | Positive | 0.5126 | Non-Allergen | Non-Toxin |
|  | PTVEGGLGF | VP1 | 1.1547 | Positive | 1.3726 | Non-Allergen | Non-Toxin |
|  | YQRANGTLA | VP1 | 0.9086 | positive | 0.4718 | Non-Allergen | Non-Toxin |
|  | RKQVNAANF | VP1 | 0.9207 | positive | 0.9583 | Non-Allergen | Non-Toxin |
|  | TELLFAPQM | VP1 | 1.0319 | Positive | 0.8241 | Non-Allergen | Non-Toxin |
|  | IVLSTIPEL | VP2 | 1.1678 | positive | 0.4270 | Non-Allergen | Non-Toxin |
|  | VYNLNPGEL | VP2 | 1.3946 | Positive | 1.6711 | Non-Allergen | Non-Toxin |
|  | LWLPQAWPW | VP2 | 1.2940 | Positive | 0.5232 | Non-Allergen | Non-Toxin |
|  | GPRIGSTTM | VP2 | 1.7585 | Positive | 0.7918 | Non-Allergen | Non-Toxin |
|  | HDEGTWVSF | VP2 | 1.0562 | Positive | 0.5537 | Non-Allergen | Non-Toxin |
|  | PEIIAATAI | VP2 | 1.0903 | Positive | 0.6054 | Non-Allergen | Non-Toxin |
|  | TEATAALAV | VP2 | 0.9892 | Positive | 0.6161 | Non-Allergen | Non-Toxin |
|  | QAIAYNRDW | VP2 | 1.4769 | positive | 0.8590 | Non-Allergen | Non-Toxin |

Table S2: Analysis of HPyV structural proteins to predict potential HTL epitopes.

| species | Epitopes | Protein | Antigenicity | Allergenicity | Toxicity | IFN | IL-4 |
| --- | --- | --- | --- | --- | --- | --- | --- |
| HPyV6 | ERWVSFGMYQTMKEN | LTAg | 0.9455 | Non-Allergen | Non-Toxin | Positive | Inducer |
|  | FERWVSFGMYQTMKE | LTAg | 0.6519 | Non-Allergen | Non-Toxin | Positive | Inducer |
|  | AAQRRVLMLESTRQD | LTAg | 0.7175 | Non-Allergen | Non-Toxin | Positive | Inducer |
|  | AQRRVLMLESTRQDL | LTAg | 0.6775 | Non-Allergen | Non-Toxin | Positive | Inducer |
|  | VAAQRRVLMLESTRQ | LTAg | 0.8394 | Non-Allergen | Non-Toxin | Positive | Inducer |
|  | KQTFERWVSFGMYQT | LTAg | 0.5040 | Non-Allergen | Non-Toxin | Positive | Inducer |
|  | QTFERWVSFGMYQTM | LTAg | 0.6457 | Non-Allergen | Non-Toxin | Positive | Inducer |
|  | TFERWVSFGMYQTMK | LTAg | 0.7156 | Non-Allergen | Non-Toxin | Positive | Inducer |
|  | GLVNLVNYAVNYNRQ | VP2 | 0.7212 | Non-Allergen | Non-Toxin | Positive | Inducer |
|  | LVNLVNYAVNYNRQW | VP2 | 0.6294 | Non-Allergen | Non-Toxin | Positive | Inducer |
|  | NLVNYAVNYNRQWET | VP2 | 0.4198 | Non-Allergen | Non-Toxin | Positive | Inducer |
|  | QGLVNLVNYAVNYNR | VP2 | 0.4098 | Non-Allergen | Non-Toxin | Positive | Inducer |
|  | IVLSTVPELSQTLFG | VP2 | 0.4173 | Non-Allergen | Non-Toxin | Positive | Inducer |
|  | NEAAIVLSTVPELSQ | VP2 | 0.5100 | Non-Allergen | Non-Toxin | Positive | Inducer |
|  | SNEAAIVLSTVPELS | VP2 | 0.5967 | Non-Allergen | Non-Toxin | Positive | Inducer |
|  | VSNEAAIVLSTVPEL | VP2 | 0.5328 | Non-Allergen | Non-Toxin | Positive | Inducer |
|  | GVVNYDMQNLPVNGN | VP2 | 0.5607 | Non-Allergen | Non-Toxin | Positive | Inducer |
|  | AAFGRFFRVHFRQRR | VP1 | 0.5924 | Non-Allergen | Non-Toxin | Positive | Inducer |
|  | AFGRFFRVHFRQRRV | VP1 | 0.4807 | Non-Allergen | Non-Toxin | Positive | Inducer |
|  | FFRVHFRQRRVKHPY | VP1 | 0.769 | Non-Allergen | Non-Toxin | Positive | Inducer |
|  | FRVHFRQRRVKHPYT | VP1 | 0.7625 | Non-Allergen | Non-Toxin | Positive | Inducer |
|  | GRFFRVHFRQRRVKH | VP1 | 0.4931 | Non-Allergen | Non-Toxin | Positive | Inducer |
|  | RFFRVHFRQRRVKHP | VP1 | 0.5138 | Non-Allergen | Non-Toxin | Positive | Inducer |
|  | AVQEVVMEQMQPSIL | VP1 | 0.4447 | Non-Allergen | Non-Toxin | Positive | Inducer |
|  | VQEVVMEQMQPSILP | VP1 | 0.6345 | Non-Allergen | Non-Toxin | Positive | Inducer |
|  | SAGYIRAQGTPAGVE | VP1 | 0.8598 | Non-Allergen | Non-Toxin | Positive | Inducer |
|  | AGYIRAQGTPAGVEG | VP1 | 0.7177 | Non-Allergen | Non-Toxin | Positive | Inducer |
|  | VGSAGYIRAQGTPAG | VP1 | 0.8446 | Non-Allergen | Non-Toxin | Positive | Inducer |
|  | RVHFRQRRVKHPYTV | VP1 | 0.9212 | Non-Allergen | Non-Toxin | Positive | Inducer |
|  | QAAFGRFFRVHFRQR | VP1 | 0.4623 | Non-Allergen | Non-Toxin | Positive | Inducer |
| HPyV7 | EQWVSYGMFQTMKEN | LTAg | 0.4580 | Non-Allergen | Non-Toxin | Positive | Inducer |
|  | QWVSYGMFQTMKENI | LTAg | 0.5279 | Non-Allergen | Non-Toxin | Positive | Inducer |
|  | AARRVLMMESTRKDL | LTAg | 0.4158 | Non-Allergen | Non-Toxin | Positive | Inducer |
|  | LAARRVLMMESTRKD | LTAg | 0.7079 | Non-Allergen | Non-Toxin | Positive | Inducer |
|  | RRVLMMESTRKDLMV | LTAg | 0.4475 | Non-Allergen | Non-Toxin | Positive | Inducer |
|  | RVLMMESTRKDLMVM | LTAg | 0.4447 | Non-Allergen | Non-Toxin | Positive | Inducer |
|  | VLAARRVLMMESTRK | LTAg | 0.6912 | Non-Allergen | Non-Toxin | Positive | Inducer |
|  | DWNFVADFAADMELT | LTAg | 1.2109 | Non-Allergen | Non-Toxin | Positive | Inducer |
|  | WNFVADFAADMELTD | LTAg | 0.8784 | Non-Allergen | Non-Toxin | Positive | Inducer |
|  | CDWNFVADFAADMEL | LTAg | 0.7160 | Non-Allergen | Non-Toxin | Positive | Inducer |
|  | MGYYMEFATEPSLCP | LTAg | 0.8124 | Non-Allergen | Non-Toxin | Positive | Inducer |
|  | RRVLMMESTRKDLMV | LTAg | 0.4475 | Non-Allergen | Non-Toxin | Positive | Inducer |
|  | FFISGSRHRVSAILN | LTAg | 0.4542 | Non-Allergen | Non-Toxin | Positive | Inducer |
|  | VFFISGSRHRVSAIL | LTAg | 0.4513 | Non-Allergen | Non-Toxin | Positive | Inducer |
|  | MELTDVLLIMGYYME | LTAg | 0.8839 | Non-Allergen | Non-Toxin | Positive | Inducer |
|  | FEPGGVVMYDTQNLP | VP2 | 0.9125 | Non-Allergen | Non-Toxin | negative | Inducer |
|  | LFEPGGVVMYDTQNL | VP2 | 0.5344 | Non-Allergen | Non-Toxin | negative | Inducer |
|  | RQNIINGANRAIEMA | VP2 | 0.5432 | Non-Allergen | Non-Toxin | negative | Inducer |
|  | QNIINGANRAIEMAP | VP2 | 0.6340 | Non-Allergen | Non-Toxin | negative | Inducer |
|  | FFRVHCRQRRIKHPY | VP1 | 0.4266 | Non-Allergen | Non-Toxin | negative | Inducer |
|  | FRVHCRQRRIKHPYT | VP1 | 0.5498 | Non-Allergen | Non-Toxin | negative | Inducer |
|  | ASLQALRKQVNAANF | VP1 | 0.6690 | Non-Allergen | Non-Toxin | negative | Inducer |
|  | QMASSGYQRANGTLA | VP1 | 0.5038 | Non-Allergen | Non-Toxin | negative | Inducer |
|  | SLQALRKQVNAANFP | VP1 | 0.5883 | Non-Allergen | Non-Toxin | negative | Inducer |
|  | RIKHPYTVDMMFRQF | VP1 | 0.5337 | Non-Allergen | Non-Toxin | negative | Inducer |

Table S3: Analysis of HPyV structural proteins to predict potential LBL epitopes.

| species | Epitopes | Protein | Antigenicity | Allergenicity | Toxicity |
| --- | --- | --- | --- | --- | --- |
| HPyV6 | IRLACKKYHPDKGGDP | LTAg | 0.7974 | Non-Allergen | Non-Toxin |
|  | SQDSKYSCTPPKKRKP | LTAg | 1.2803 | Non-Allergen | Non-Toxin |
|  | PSCLHDYLSHATLGNK | LTAg | 0.6503 | Non-Allergen | Non-Toxin |
|  | RKPNPAPNDFPSCLHD | LTAg | 0.5204 | Non-Allergen | Non-Toxin |
|  | FQEGSKKEECDWNQVA | LTAg | 0.7703 | Non-Allergen | Non-Toxin |
|  | TGAILYCITPRRHRVS | LTAg | 0.7895 | Non-Allergen | Non-Toxin |
|  | SCTPPKKRKPNPAPND | LTAg | 1.2515 | Non-Allergen | Non-Toxin |
|  | GVEITQLLGAVAWLDC | LTAg | 0.5416 | Non-Allergen | Non-Toxin |
|  | SSEVRPPPQYGSPGWE | LTAg | 1.0311 | Non-Allergen | Non-Toxin |
|  | GMYQTMKENILAGIDP | LTAg | 0.5054 | Non-Allergen | Non-Toxin |
|  | CVPVNMERKHLNKVSQ | LTAg | 0.7237 | Non-Allergen | Non-Toxin |
|  | YQSRGAPYTFTDTLDA | VP1 | 0.6081 | Non-Allergen | Non-Toxin |
|  | VGYAGNPTLSDAYSQQ | VP1 | 0.7373 | Non-Allergen | Non-Toxin |
|  | PSKENQPSVAGIKATR | VP1 | 1.3222 | Non-Allergen | Non-Toxin |
|  | VAGIKATRKQVTAANF | VP1 | 1.2442 | Non-Allergen | Non-Toxin |
|  | DVIGINPDPERMNVAA | VP1 | 0.6634 | Non-Allergen | Non-Toxin |
|  | YIRAQGTPAGVEGSQM | VP1 | 0.7140 | Non-Allergen | Non-Toxin |
|  | AGSSLCYTLAVVNLPE | VP1 | 0.5917 | Non-Allergen | Non-Toxin |
|  | PSILPTTLEGAIGYSP | VP1 | 0.8619 | Non-Allergen | Non-Toxin |
|  | PTLSDAYSQQRSVQAA | VP1 | 0.6159 | Non-Allergen | Non-Toxin |
|  | YLTSADMLGMVGYAGN | VP1 | 0.7688 | Non-Allergen | Non-Toxin |
|  | SWGGAGRGLPDWLINM | VP2 | 0.4555 | Non-Allergen | Non-Toxin |
|  | GVIYSNYNPGELYKAP | VP2 | 0.4050 | Non-Allergen | Non-Toxin |
|  | PEIIAAAAVGGGEALE | VP2 | 0.5846 | Non-Allergen | Non-Toxin |
|  | ASRELGALLSRVRETV | VP2 | 0.7231 | Non-Allergen | Non-Toxin |
|  | SNKKRRSGGYGNSATF | VP2 | 0.7625 | Non-Allergen | Non-Toxin |
| HPyV7 | VRLASKKYHPDKGGDP | LTAg | 0.5666 | Non-Allergen | Non-Toxin |
|  | TIKIKEMLSYLVENTP | LTAg | 0.4447 | Non-Allergen | Non-Toxin |
|  | DSKYSATPPKQKKPNP | LTAg | 1.1870 | Non-Allergen | Non-Toxin |
|  | SDEVRPPPPYGSPAWD | LTAg | 0.5795 | Non-Allergen | Non-Toxin |
|  | SSSDEEEPAASASVNP | LTAg | 0.5965 | Non-Allergen | Non-Toxin |
|  | GVEITQLIGAVAWLDC | LTAg | 0.5564 | Non-Allergen | Non-Toxin |
|  | NPEEGCSQDSKYSATP | LTAg | 1.0140 | Non-Allergen | Non-Toxin |
|  | PSGVGMANLDNMRDYL | LTAg | 0.7463 | Non-Allergen | Non-Toxin |
|  | VVIEDVKGTPLPNTDL | LTAg | 0.9250 | Non-Allergen | Non-Toxin |
|  | KKPNPAPQDFPECLSE | LTAg | 0.6059 | Non-Allergen | Non-Toxin |
|  | PRVIRKGGVEVLDTVP | VP1 | 0.4197 | Non-Allergen | Non-Toxin |
|  | GGLGFAPTSKFLIQNG | VP1 | 0.7276 | Non-Allergen | Non-Toxin |
|  | PATIPPTVEGGLGFAP | VP1 | 1.1122 | Non-Allergen | Non-Toxin |
|  | TGLPGLPTLSADYSNQ | VP1 | 0.5053 | Non-Allergen | Non-Toxin |
|  | GGVTPPVVSYGNQSTT | VP1 | 0.6090 | Non-Allergen | Non-Toxin |
|  | PLTEETQYKVEAVLLP | VP1 | 0.6824 | Non-Allergen | Non-Toxin |
|  | VVMYDTQNLPLSGNND | VP2 | 0.47 | Non-Allergen | Non-Toxin |
|  | KRKWTHTETSQSDKKR | VP2 | 0.5 | Non-Allergen | Non-Toxin |
|  | TWVSFQGEEGNTPQYT | VP2 | 0.49 | Non-Allergen | Non-Toxin |
|  | DQRGGFHDEGTWVSFQ | VP2 | 1.1 | Non-Allergen | Non-Toxin |


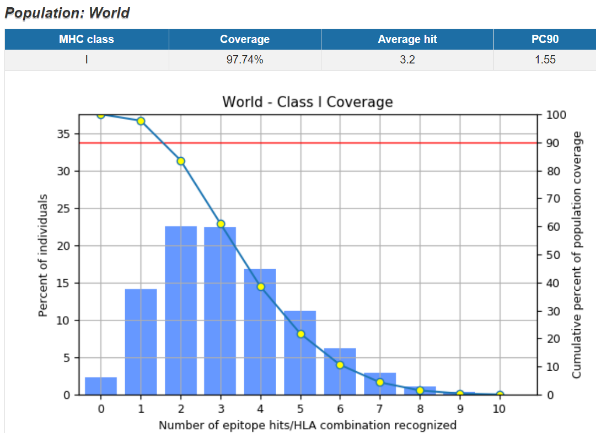

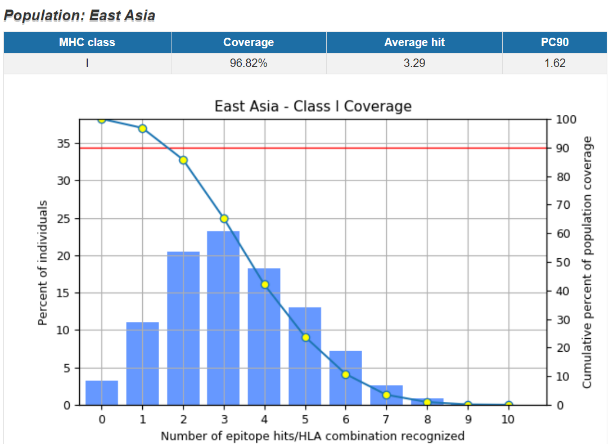


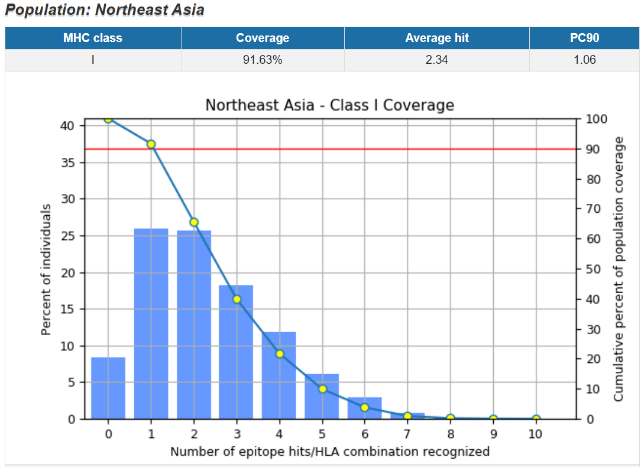

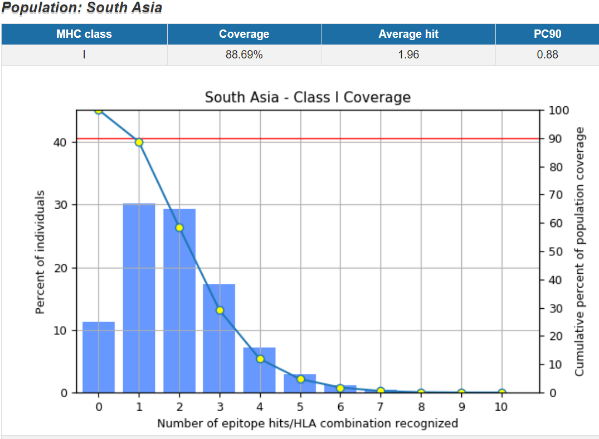


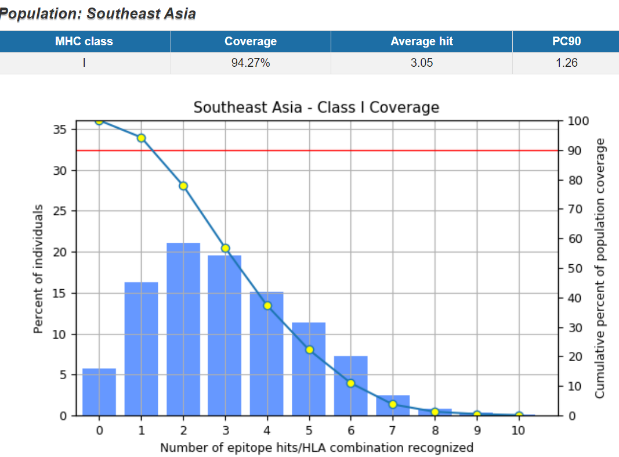

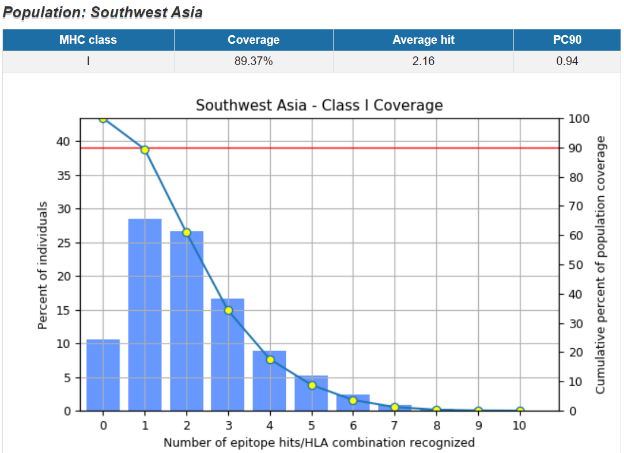


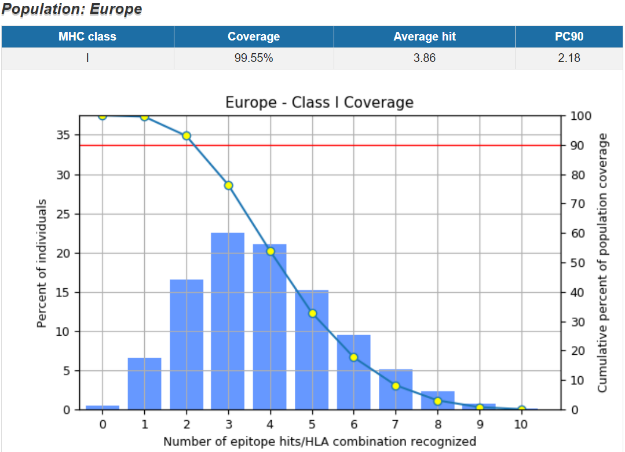

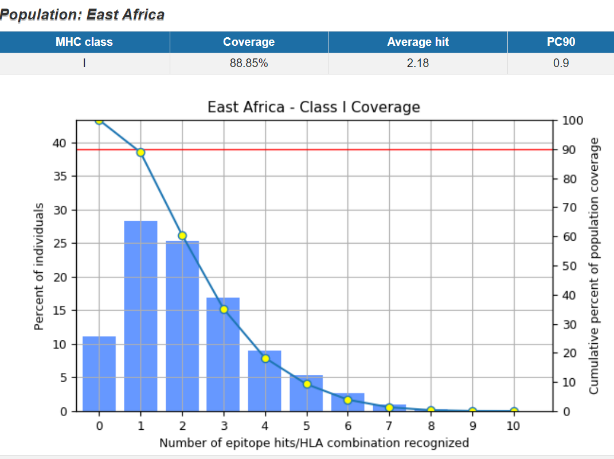


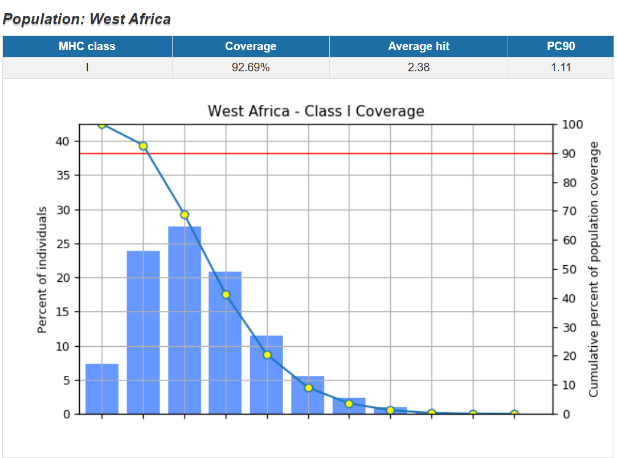

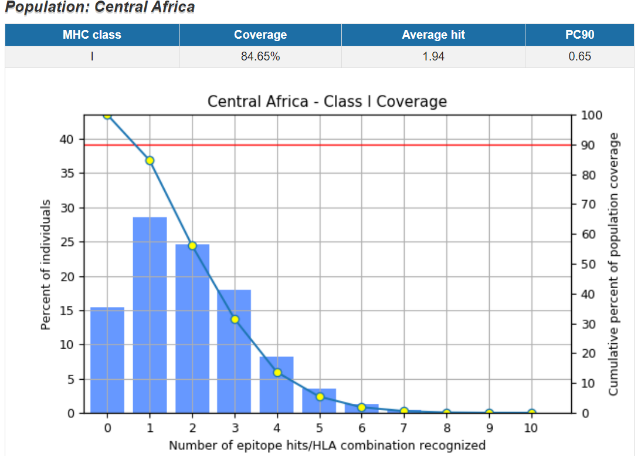


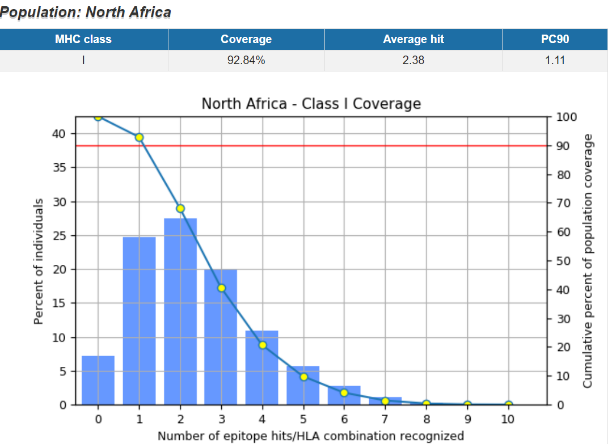

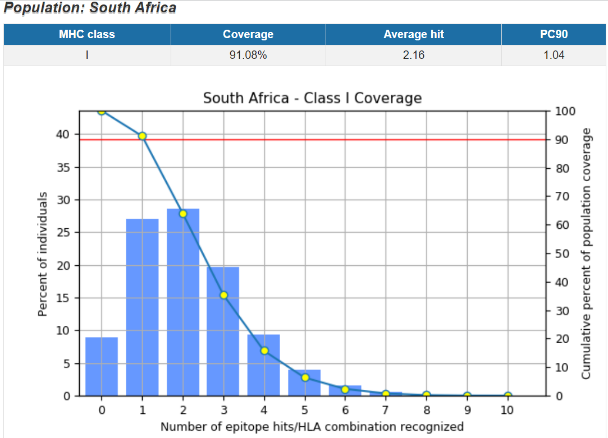


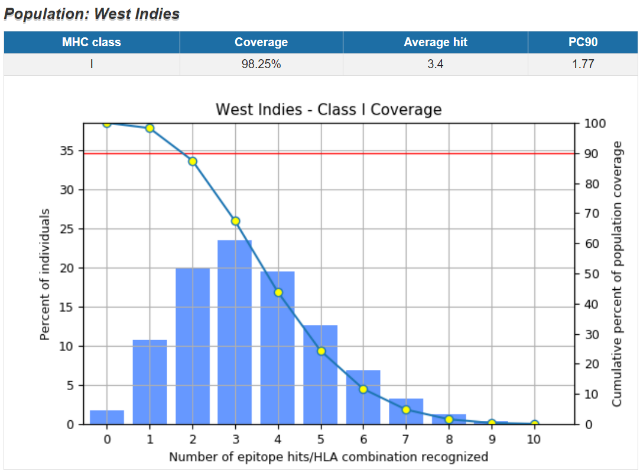

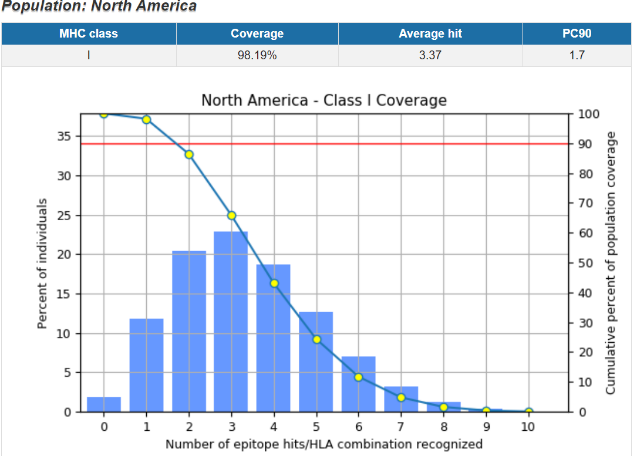


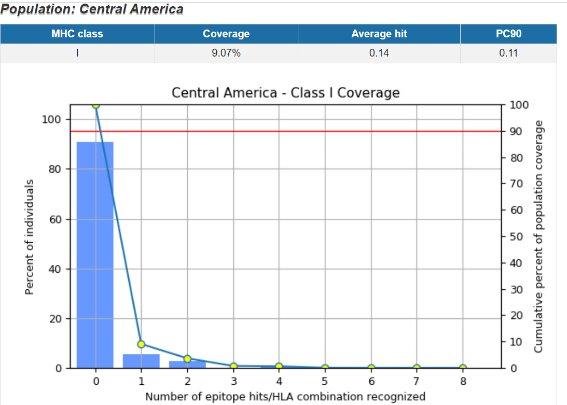

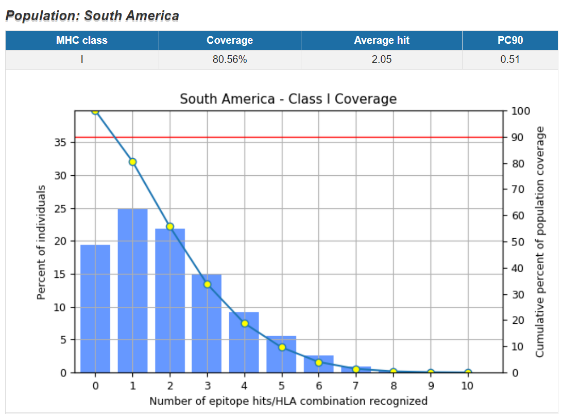


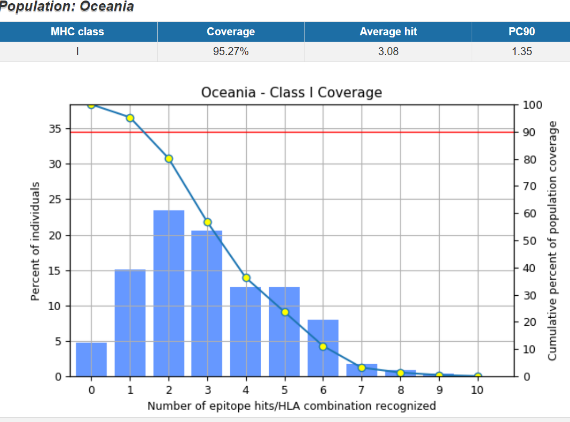


Fig S1: Evaluation of population coverage of final epitopes obtained from MHCI restriction data by the IEDB analysis resource.


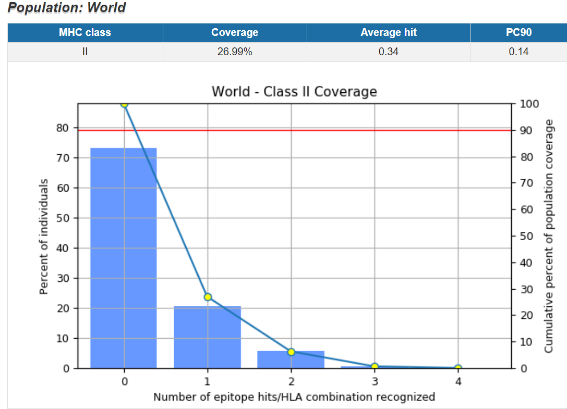

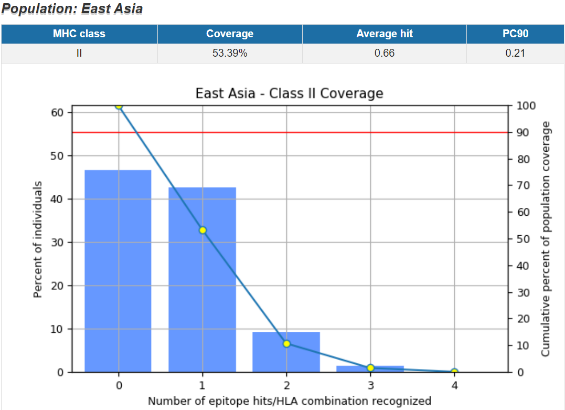


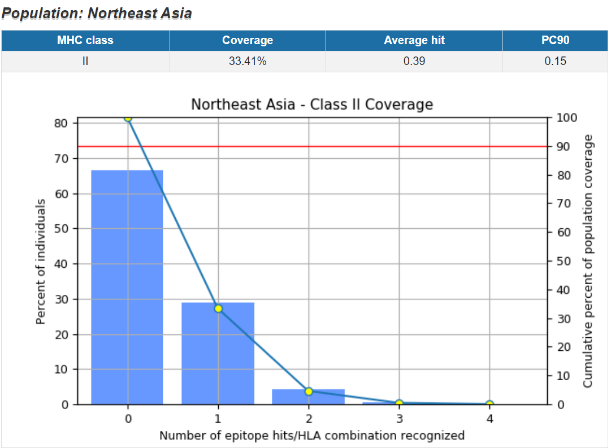

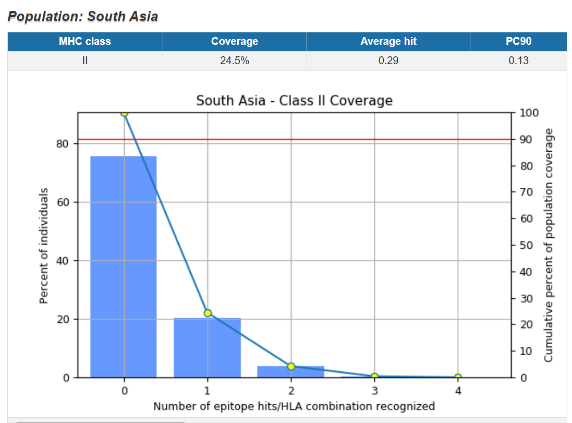


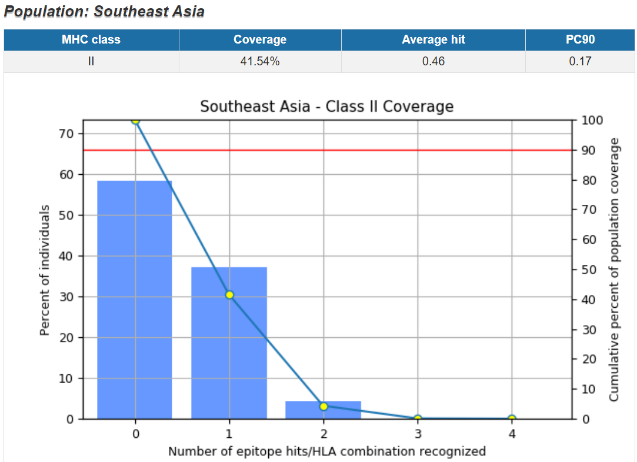

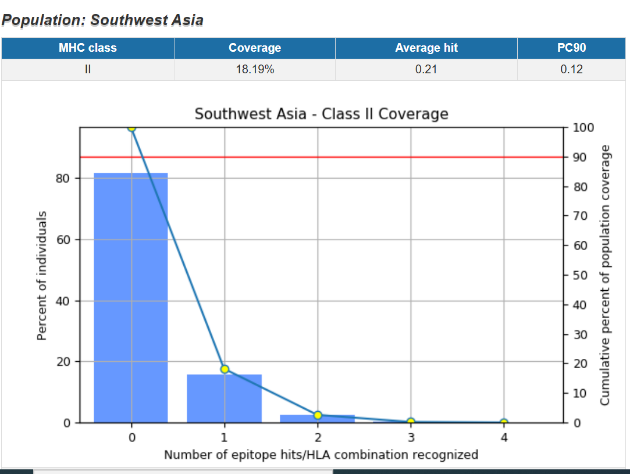


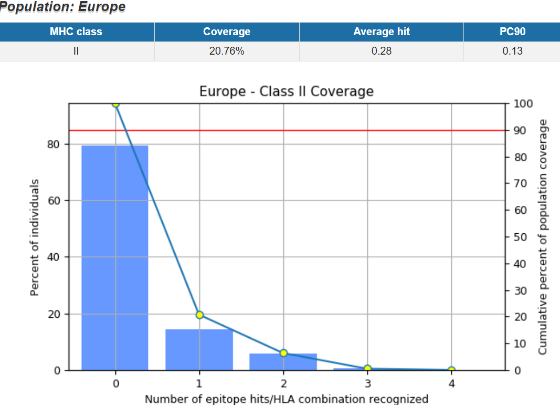

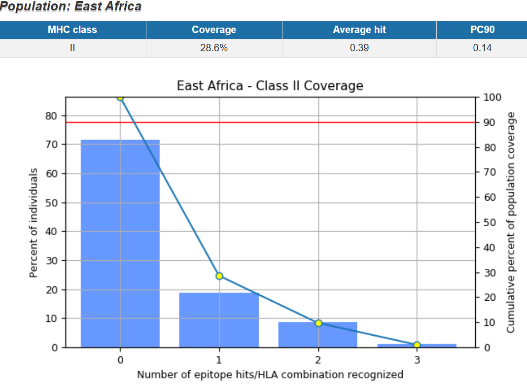


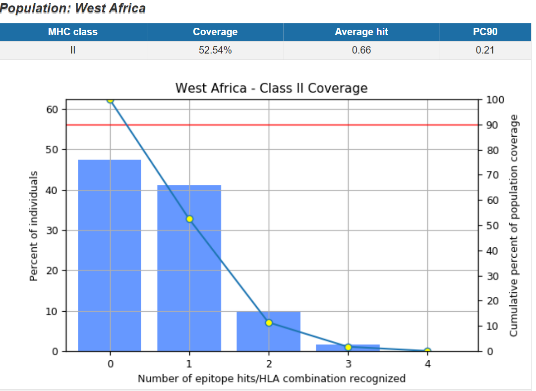

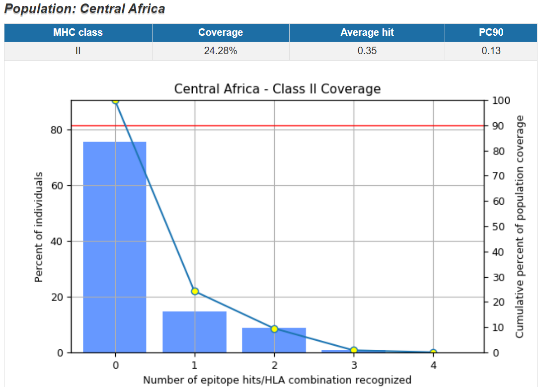


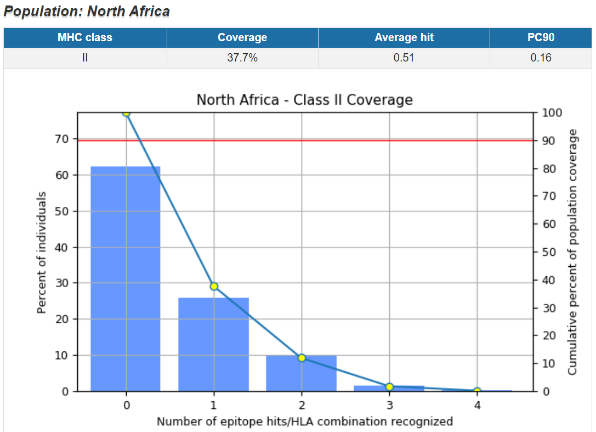

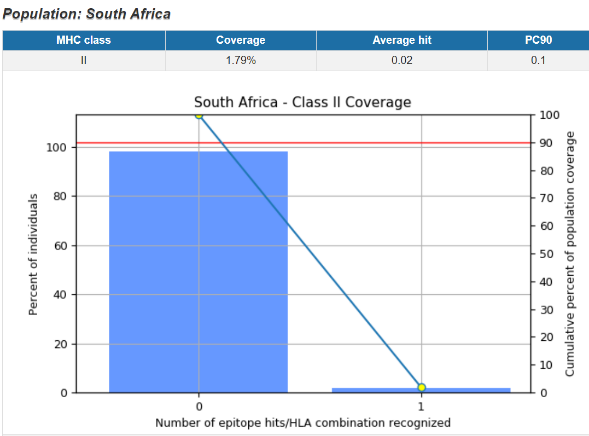


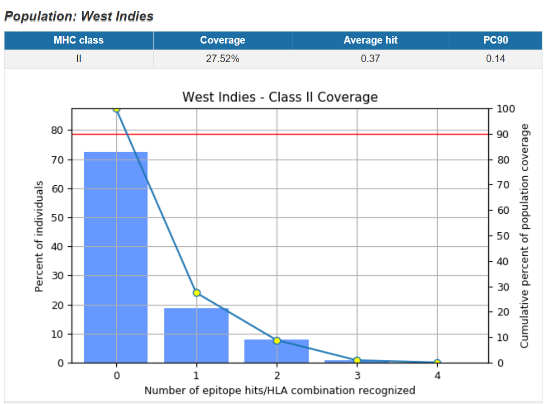

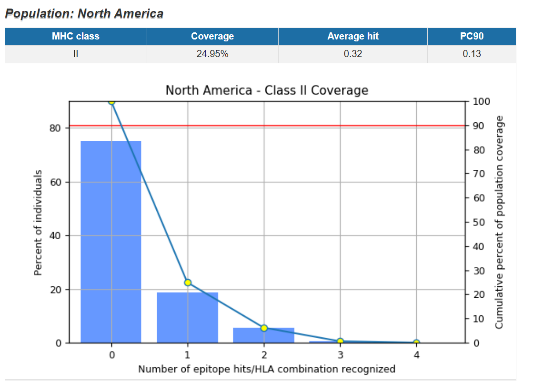


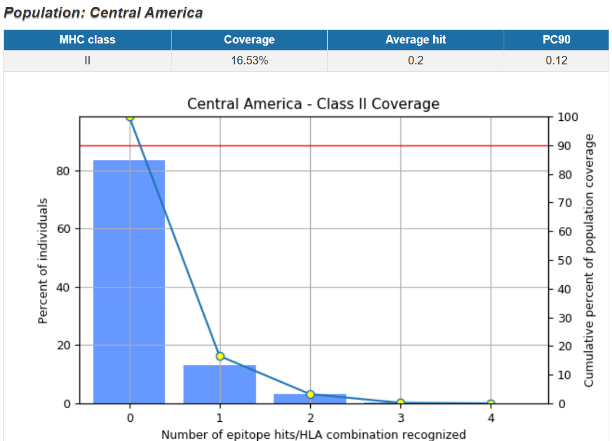

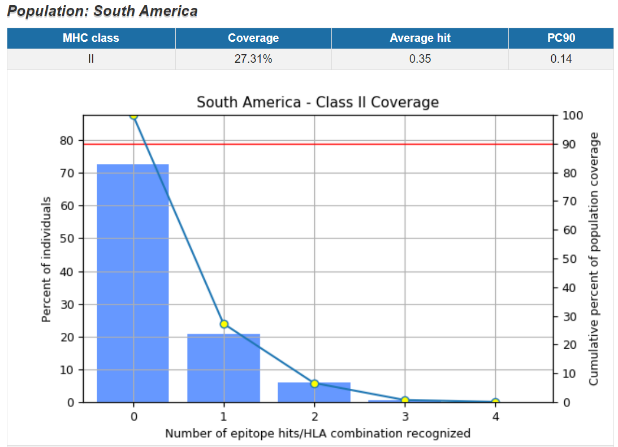


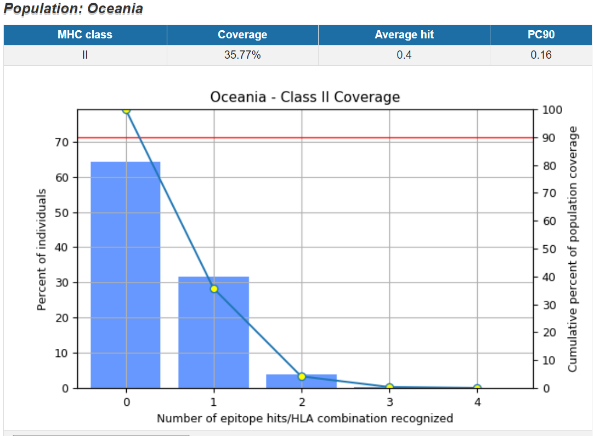


Fig S2: Evaluation of population coverage of final epitopes obtained from MHCII restriction data by the IEDB analysis resource.


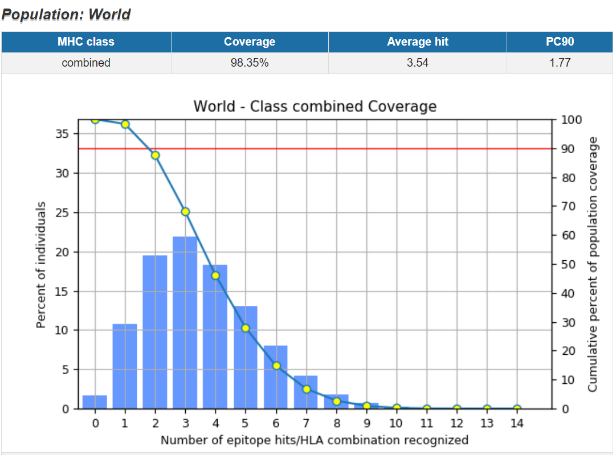

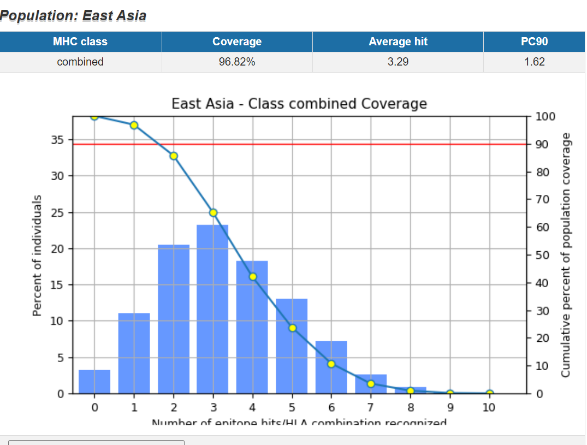


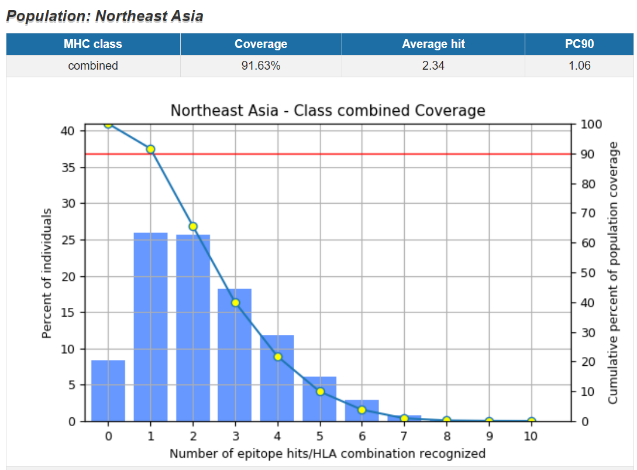

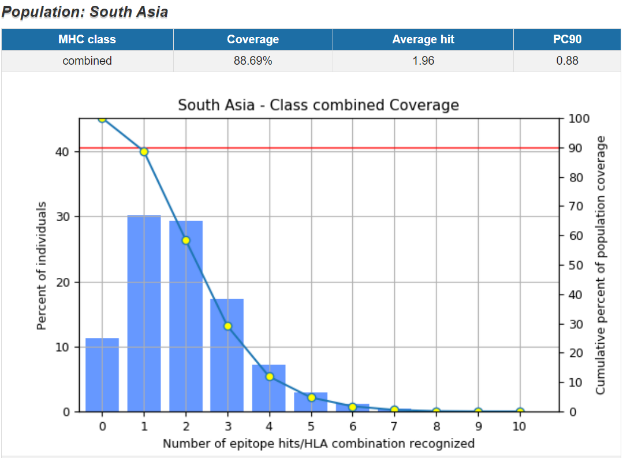


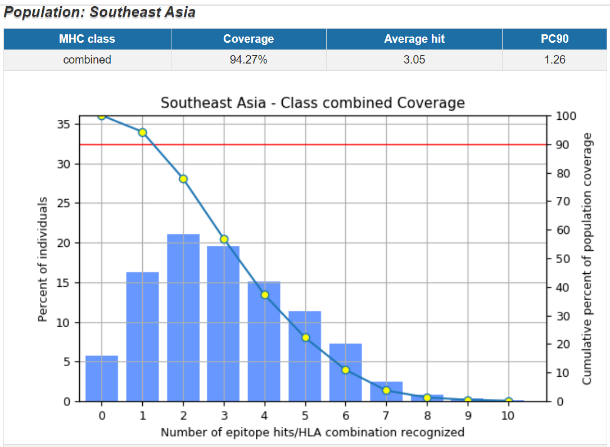

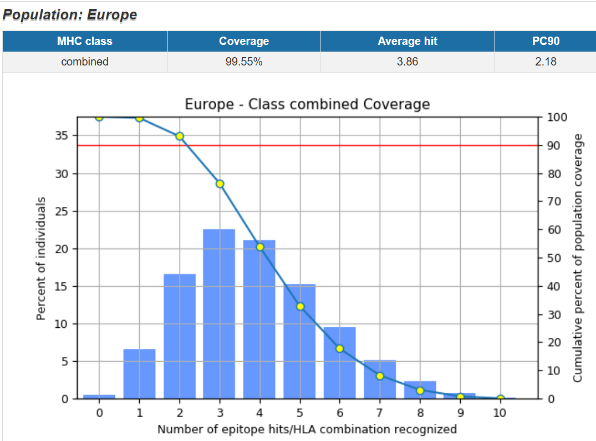


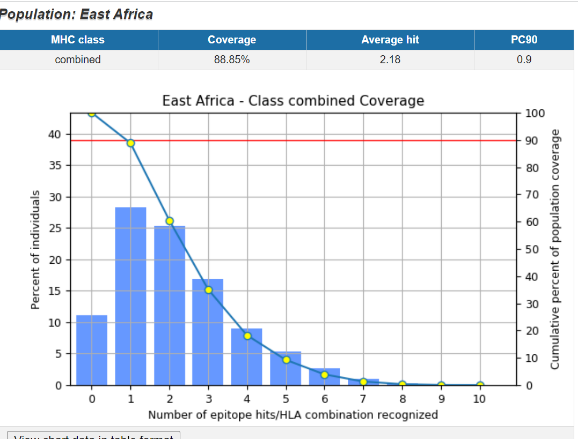

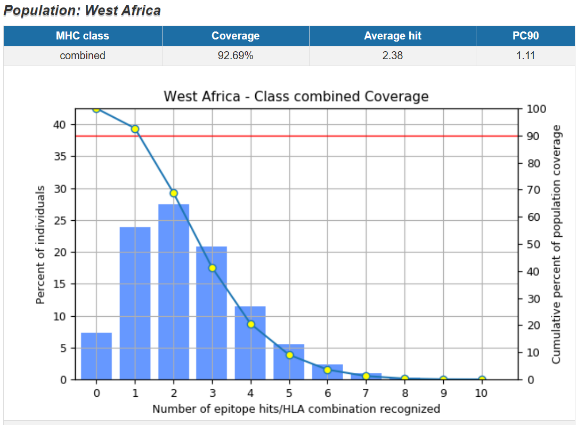


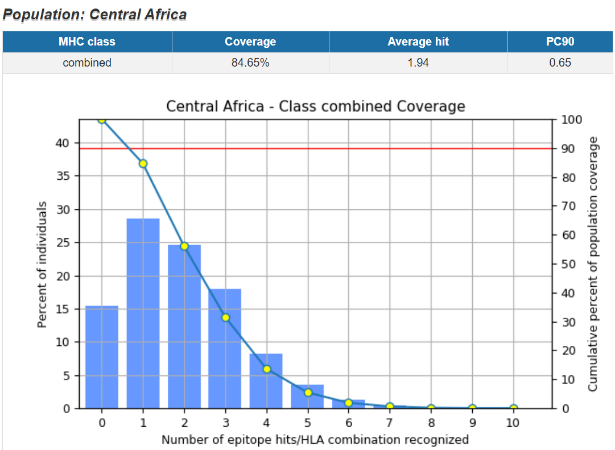

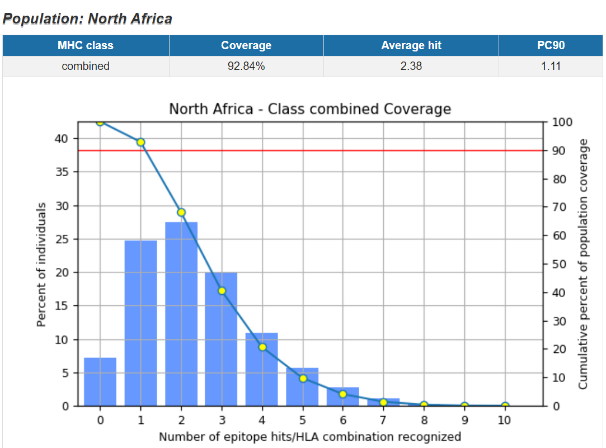


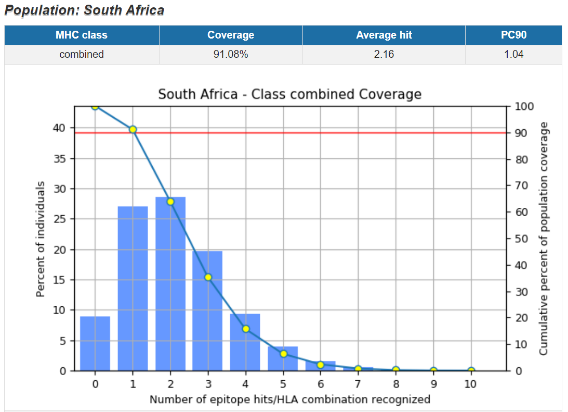

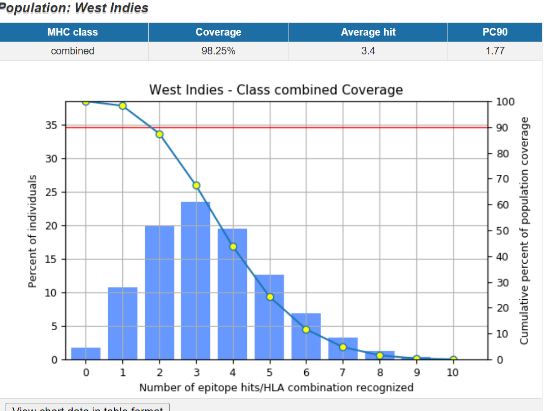


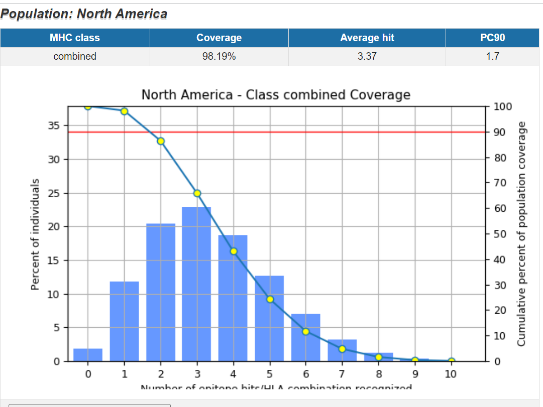

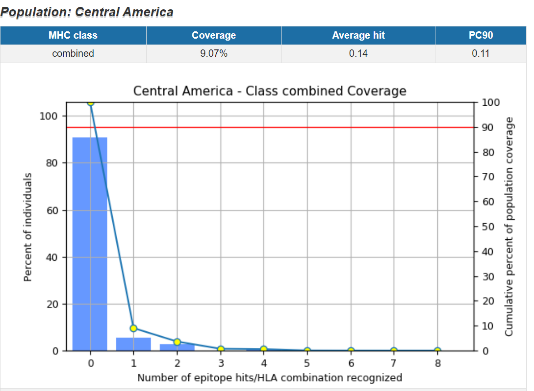


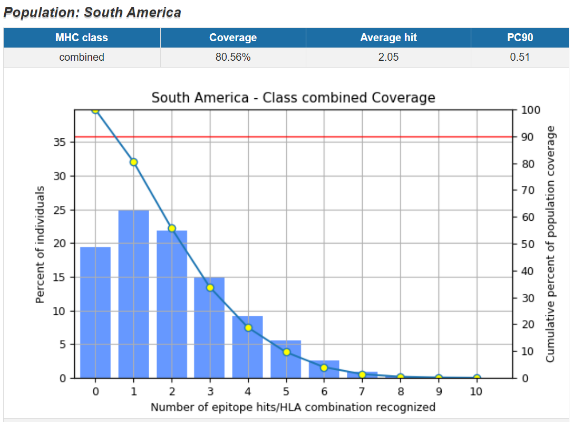

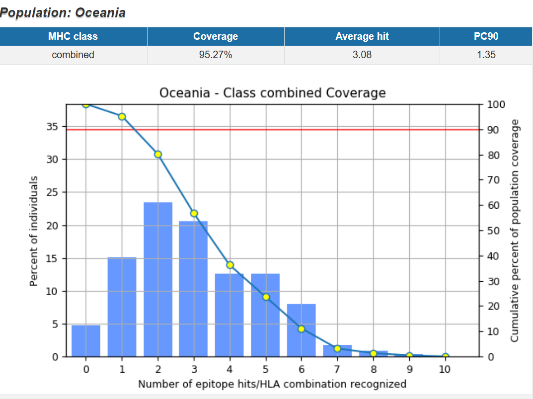


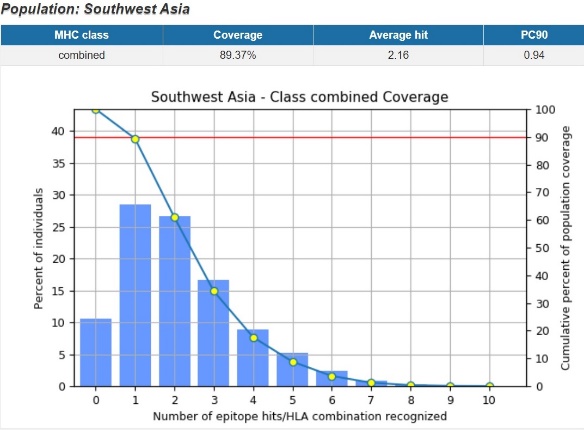


Fig S3: Evaluation of population coverage of final epitopes obtained from composed MHCI and MHCII restriction data by the IEDB analysis resource.
